# Overcoming the blood–brain barrier in Alzheimer's disease: translational perspectives on advanced drug delivery platforms

**DOI:** 10.3389/fnins.2026.1810486

**Published:** 2026-05-14

**Authors:** Napaporn Roamcharern, Ruedeemars Yubolphan

**Affiliations:** 1 Strathclyde Institute of Pharmacy and Biomedical Sciences, University of Strathclyde, Glasgow, United Kingdom; 2 Department of Pharmacology, Faculty of Medicine, Chiang Mai University, Chiang Mai, Thailand

**Keywords:** central nervous system delivery, nanomedicines, nanoparticles, neurodegenerative disorder, nose-to-brain delivery

## Abstract

Alzheimer's disease (AD) is the leading cause of dementia worldwide and represents a growing public health challenge in aging societies. Despite extensive research efforts, currently approved therapies provide only limited symptomatic benefit and do not halt disease progression. A major obstacle to effective treatment is the blood–brain barrier (BBB), which severely restricts the brain delivery of most therapeutic agents. Nanoparticle-based drug delivery systems have emerged as a promising strategy to overcome BBB-related limitations by enabling precise control over physicochemical properties such as size, surface characteristics, and material composition. These properties can improve drug solubility, stability, pharmacokinetics, and targeted brain accumulation while reducing systemic toxicity. However, efficient BBB penetration and clinically feasible translation remain major challenges. This review summarizes key design principles for nanoparticles intended for AD therapy and highlights representative platforms with translational considerations, particularly lipid-based and polymer-based nanoparticles. In addition, alternative delivery strategies—including nose-to-brain nanoparticle systems and nanoparticles exploiting receptor-mediated and adsorptive-mediated transcytosis, as well as synaptic dysfunction targeting—are discussed. Collectively, this review outlines current advances and future directions for nanoparticle-mediated therapeutic delivery in AD.

## 1 Introduction

Alzheimer's disease (AD) is recognized as a neurodegenerative disorder that accounts for approximately 60%−70% of dementia cases worldwide. Clinically, AD is characterized by a progressive and irreversible decline in cognitive function, including impairments in memory, executive function, language, and behavior, ultimately leading to complete dependence and premature death (Scheltens et al., 2021). With rapid global population aging, the prevalence of AD is projected to rise dramatically, posing an increasing burden on healthcare systems, caregivers, and societies.

More than 55 million individuals are currently living with dementia globally, a number expected to exceed 130 million by 2050 (Tahami Monfared et al., 2022). The socioeconomic impact of AD is substantial, encompassing direct medical costs, long-term care, and indirect costs related to caregiver burden and lost productivity. Despite decades of intensive research, therapeutic progress in AD has remained limited. Approved pharmacological treatments, such as acetylcholinesterase inhibitors and the *N*-methyl-d-aspartate (NMDA) receptor antagonist memantine, provide only modest symptomatic relief and do not alter disease progression; thereby, AD has emerged as a particularly difficult-to-treat disorder (Tenchov et al., 2024).

Due to critical limitations in the treatment of AD, the blood–brain barrier (BBB) restricts the entry of approximately 98% of the US Food and Drug Administration (FDA)-approved small-molecule drugs as well as most large-molecule therapeutics (Pardridge, 2005). More recently, disease-modifying therapies targeting amyloid-β (Aβ), including monoclonal antibodies, have demonstrated the ability to reduce amyloid burden (Monteiro et al., 2023). However, their clinical benefits remain modest and variable, and their use is associated with safety concerns such as amyloid-related imaging abnormalities (ARIA), costly treatment, and invasive administration routes. These limitations underscore the urgent need for more effective and safer therapeutic strategies (Hardy, 2025).

Nanoparticles have gained substantial attention in pharmaceutical research as an advanced drug delivery system (DDS), owing to their capacity for rational optimization and customization–through size (Kulkarni and Feng, 2013; Brown et al., 2020; Song et al., 2021; De Lima and Mortari, 2022), morphology (Brown et al., 2020; Song et al., 2021), surface charge and modification (Kulkarni and Feng, 2013; Song et al., 2021; De Lima and Mortari, 2022), and material/composition selection (Brown et al., 2020; De Lima and Mortari, 2022)–to meet specific therapeutic objectives. The BBB restriction also applies to nanoparticles, thereby hindering targeted drug delivery to specific pathological regions of the brain within the central nervous system (CNS) (Alyautdin et al., 2014; Pandit et al., 2020; Wu et al., 2023). To overcome this significant challenge, various drug nanoparticle types featuring advanced strategic designs tailored to their unique physicochemical properties have emerged (Song et al., 2021; De Lima and Mortari, 2022).

Engineered nanoparticles are designed to confer physicochemical properties that can improve drug solubility, stability, biodistribution, and pharmacokinetic behavior (Chamundeeswari et al., 2019). These features may prolong drug half-life and reduce systemic toxicity, thereby enhancing drug delivery to pathological sites within the central nervous system (Sairam et al., 2023). This targeted delivery can significantly reduce dosing frequency (Wang et al., 2021; Cai et al., 2023), while the nanoparticle itself simultaneously demonstrates excellent biocompatibility (Sathish Sundar et al., 2016; De et al., 2022) and controllable biodegradability (Sathish Sundar et al., 2016; De et al., 2022).

This review article highlights the key properties required for the development of drug nanoparticles and discusses exemplary nanoparticle platforms with strong potential for overcoming the BBB, particularly lipid-based and polymeric nanoparticles. We also discuss the alternative delivery approaches, such as the nose-to-brain nanoparticle systems, which bypass the BBB restriction, along with other strategically designed nanoparticles employing receptor-mediated transcytosis (RMT), adsorptive-mediated transcytosis (AMT), and synaptic dysfunction targeting.

## 2 Alzheimer's disease

### 2.1 Pathophysiology of Alzheimer's disease

AD is a multifactorial disorder driven by interconnected molecular and cellular pathological processes. The classical neuropathological hallmarks of AD include extracellular amyloid plaques composed of Aβ peptides and intracellular neurofibrillary tangles formed by hyperphosphorylated tau protein (Liu et al., 2024; Tenchov et al., 2024). These pathological features are accompanied by synaptic loss, chronic neuroinflammation, mitochondrial dysfunction, oxidative stress, and progressive neuronal degeneration (Wang et al., 2020; Mani et al., 2025).

Aβ peptides are produced by the sequential cleavage of amyloid precursor protein (APP) by β-secretase (BACE1) and γ-secretase. Among various Aβ species, Aβ_1 − 42_ is particularly aggregation-prone and neurotoxic. Increasing evidence suggests that soluble Aβ oligomers, rather than insoluble plaques, are major contributors to synaptic dysfunction and cognitive decline in AD, although their precise pathogenic role remains under active investigation and debate (Cleary et al., 2005; Selkoe, 2008; Yang et al., 2017; Walsh and Selkoe, 2020; Fedele, 2023). Effective therapeutic intervention, therefore, requires adequate drug concentrations at synaptic and perisynaptic sites, highlighting the importance of precise CNS drug delivery.

Tau pathology represents a second major axis of AD progression and correlates more closely with cognitive impairment than amyloid burden. Hyperphosphorylated tau dissociates from microtubules, disrupts axonal transport, and forms neurofibrillary tangles. In addition, pathological tau has been shown to spread between neurons through seeded, prion-like propagation, in which misfolded tau can induce templated aggregation in connected cells, thereby contributing to the progression of tau pathology across brain regions. This mechanism suggests that effective therapies may require intracellular delivery and interruption of cell-to-cell transmission, both of which present substantial delivery challenges (Lasagna-Reeves et al., 2012; Wang et al., 2025b).

Neuroinflammation further amplifies disease progression. Activated microglia and astrocytes release proinflammatory cytokines, chemokines, and reactive oxygen species, contributing to synaptic dysfunction and neuronal death (Thakur et al., 2023). Genetic associations with immune-related genes, such as TREM2, further underscore the critical role of neuroimmune pathways in AD (Raulin et al., 2022). Given the growing therapeutic interest in TREM2-mediated modulation of microglial function, nanoparticle-based systems may also provide opportunities to enhance the brain delivery of TREM2-targeted biologics or nucleic acid therapeutics (Li et al., 2024; Qi et al., 2025). In parallel, mitochondrial dysfunction and oxidative stress exacerbate neuronal vulnerability and energy failure (Zheng and Wang, 2025). Together, these diverse yet interconnected mechanisms indicate that AD is unlikely to be effectively treated through single-target approaches and instead requires delivery strategies capable of addressing multiple pathological pathways.

### 2.2 Blood–brain barrier: structure and challenges for Alzheimer's therapy

A central obstacle to effective AD treatment is the BBB, a highly selective interface that governs molecular exchange between systemic circulation and CNS. The BBB is constructed by specialized brain microvascular endothelial cells joined by tight junctions and supported by pericytes, astrocytic end-feet, and the basement membrane, collectively constituting the neurovascular unit (Kaya and Ahishali, 2021). While the BBB is essential for maintaining CNS homeostasis, it severely restricts drug delivery. Small-molecule drugs and nearly all biologics—including peptides, proteins, antibodies, and nucleic acids—fail to cross the BBB at therapeutically relevant levels. In addition, efflux transporters such as *P*-glycoprotein (*P*-gp) and breast cancer resistance protein (BCRP) actively remove many therapeutic agents from the brain (Kadry et al., 2020). This is also clinically relevant for AD drug development, as transporter-mediated efflux can limit CNS exposure of candidate therapeutics; for example, donepezil has been reported to interact with both *P*-gp and BCRP, underscoring how BBB efflux biology may influence brain drug availability and therapeutic response (Asante and Barger, 2025).

In AD, BBB integrity is often altered due to vascular dysfunction, inflammation, and cerebral amyloid angiopathy (Cai et al., 2018; Alkhalifa et al., 2023). However, available neuroimaging evidence suggests that BBB disruption in AD is typically subtle, region-specific, and heterogeneous, rather than a uniform barrier opening that would reliably permit drug penetration across patients or brain regions (Sweeney et al., 2018; Chagnot et al., 2021). At the same time, BBB dysfunction may further exacerbate disease progression by impairing Aβ clearance and promoting neuroinflammation. Consequently, BBB dysfunction in AD represents both a pathological contributor and a major therapeutic challenge.

### 2.3 Limitations of current Alzheimer's disease therapies

Despite significant advances in understanding AD pathogenesis, current therapeutic options remain inadequate. A fundamental limitation of existing AD treatments is insufficient drug exposure in the brain. Poor BBB penetration, rapid systemic metabolism, and dose-limiting peripheral toxicity often necessitate high systemic doses, increasing the risk of adverse effects while yielding limited CNS efficacy (Passeri et al., 2022). Moreover, most current therapies focus on single pathological targets, despite the multifactorial nature of AD. Chronic treatment requirements further complicate therapy, particularly in elderly patients with multiple comorbidities who are highly susceptible to adverse drug reactions and drug–drug interactions (Cummings et al., 2019; Singh et al., 2024). These limitations emphasize that therapeutic failure in AD is frequently driven not only by insufficient target engagement but also by ineffective drug delivery.

## 3 Rationale for drug delivery system in Alzheimer's disease: targeting the blood–brain barrier

Drug delivery systems (DDS) offer several advantages highly relevant to AD, including protection of therapeutic agents from degradation, improved solubility of poorly water-soluble drugs, prolonged circulation time, and the ability to engineer surface properties for active targeting (Singh et al., 2024). Importantly, DDS enables multifunctional platforms capable of integrating therapeutic, targeting, and diagnostic functions—an approach particularly suited to the complexity of AD pathology (Nunes et al., 2022; Singh et al., 2025).

A primary rationale for DDS in AD is enhanced CNS penetration. Nanoparticles can be engineered to cross the BBB via receptor-mediated transcytosis or adsorptive-mediated transcytosis, or to bypass the BBB altogether through alternative routes such as intranasal delivery, as described in-depth in Section 4.3. By shielding drugs from peripheral metabolism and efflux transporters, DDS increase the fraction of administered drug reaching the brain while reducing systemic exposure (Roghani et al., 2024). Furthermore, DDS allows targeted delivery to specific cellular and subcellular compartments, including neurons, microglia, astrocytes, and the neurovascular unit. Such precise targeting improves therapeutic specificity and supports strategies aimed at modulating neuroinflammation, tau pathology, mitochondrial dysfunction, and synaptic failure (Shekho et al., 2024).

Beyond BBB penetration, the selection of DDS in AD should also consider disease-specific pathological features that may affect therapeutic access and efficacy. Changes in BBB integrity, endothelial receptor and transporter function, vascular dysfunction, amyloid burden, and neuroinflammatory status can influence nanoparticle penetration, biodistribution, cellular uptake, and target engagement (Alkhalifa et al., 2023). For example, receptor-mediated nanoparticles may depend on the expression and function of endothelial transport receptors, while neuroinflammatory changes may alter barrier permeability and uptake behavior (Haqqani et al., 2024). Therefore, rational DDS design in AD should align carrier properties and targeting strategies with the pathological context of the diseased brain.

**Figure 1:**
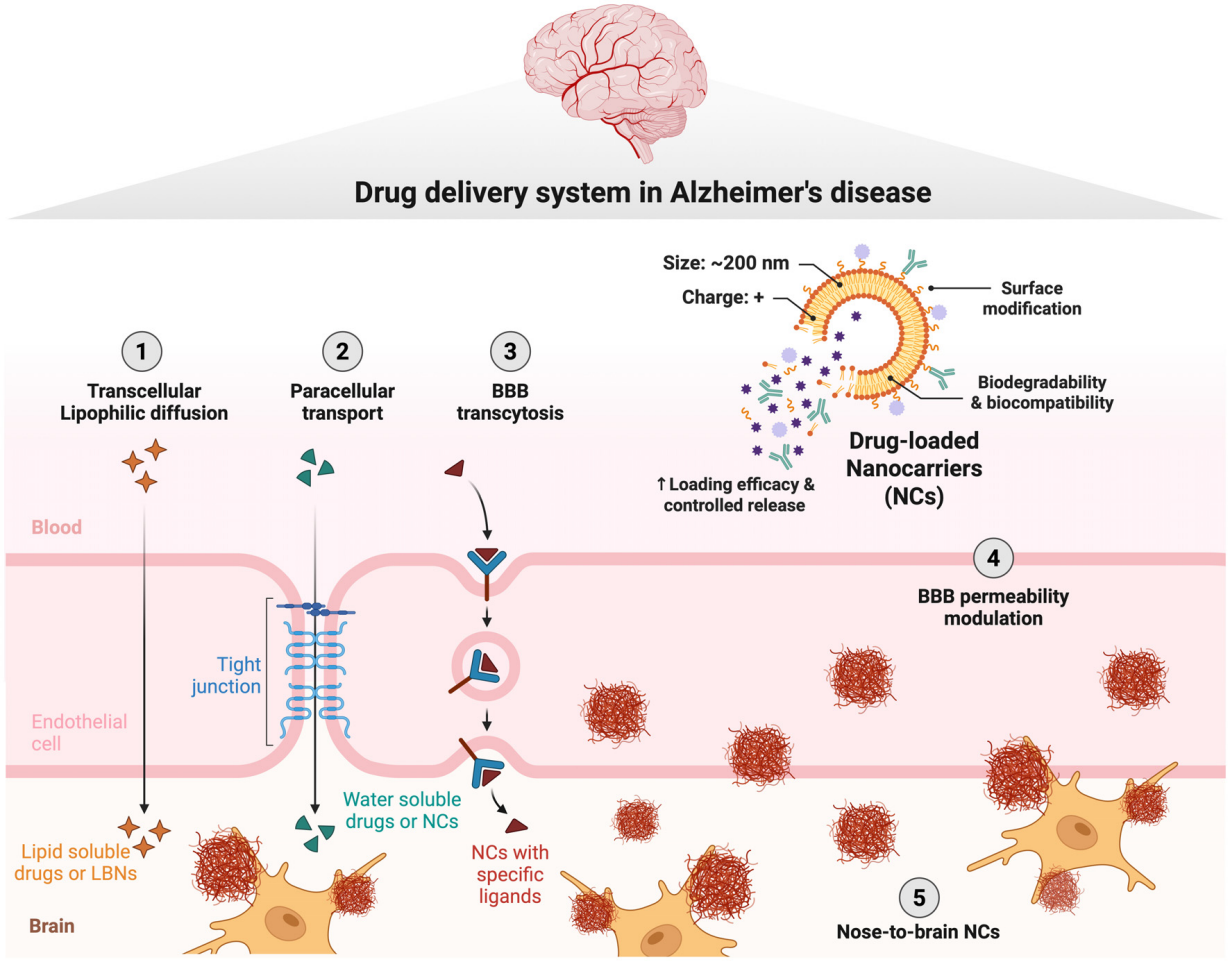
Drug delivery strategies for overcoming the blood–brain barrier in Alzheimer's disease. Schematic illustration of key transport pathways and nanoparticle-based approaches for enhancing drug delivery across the blood–brain barrier (BBB): (1) transcellular lipophilic diffusion permits passive transport of small lipid-soluble molecules across endothelial cells; (2) paracellular transport allows the limited passage of hydrophilic compounds through tight junctions; (3) BBB transcytosis allows a transportation of drug delivery system across the BBB; (4) BBB permeability modulation promotes transient barrier opening to enhance drug penetration; (5) nose-to-brain delivery bypasses the BBB through intranasal pathways, enabling direct transport to the central nervous system. Figure created with BioRender.com and adapted from the “Solute Transfer Across blood–brain Barrier” template by Nick Delrose (BioRender). Licensed under CC BY 4.0 (https://creativecommons.org/licenses/by/4.0/).

Therefore, rational DDS design in AD should align carrier properties and targeting strategies with the pathological context of the diseased brain. In this way, DDS-based approaches help address a central bottleneck in AD therapeutics: the gap between promising pharmacological targets and effective brain delivery. By improving CNS bioavailability, enabling more precise targeting, and reducing systemic toxicity, these systems offer a promising framework for enhancing the translational success of both existing and emerging AD therapies (Figure 1).

## 4 Drug delivery for Alzheimer's disease: formulations, ideal properties, systemically relevant effects, and delivery strategies of nanoparticles

### 4.1 Ideal physicochemical properties of nanoparticles for Alzheimer's disease

#### 4.1.1 Size and charge

Basically, for the treatment of AD and other central nervous system (CNS) disorders, nanoparticles with diameters of ~200 nm are considered optimal for the systemic pathway, as larger nanoparticles are more likely to be cleared by the reticuloendothelial system (RES) (Cao et al., 2024; Markova et al., 2022). In contrast, nanoparticles with diameters ranging from 100 to 700 nm are favorable for nose-to-brain delivery, an alternative route that bypasses the BBB (Markova et al., 2022). However, critically, as research on transcytosis efficiency has progressed, nanoparticles with smaller diameters (< 200 nm) have emerged as a promising size range for enhancing BBB penetration, whereas those with diameters >200 nm exhibit reduced permeability across the BBB (Betzer et al., 2017; Ceña and Játiva, 2018; Hersh et al., 2022). For instance, a size-dependent study of surface-modified poly(lactic-co-glycolic acid) (PLGA) nanoparticles demonstrated that particles with the smallest size (60 nm) exhibited the highest brain accumulation compared with larger particles, suggesting that smaller nanoparticles traverse the BBB more efficiently than larger particles (Meng et al., 2022).

Naturally, nanoparticles interact with numerous biological components in the bloodstream, which influences their biologically relevant activities. Morphology and surface charge are key variables that can be tailored to enhance these desired functions. For example, spherical nanoparticles tend to maximize circulation time and improve biodistribution, while low-surface-charge nanoparticles promote optimal interactions both among themselves and with biological components (Cao et al., 2024; Pandey et al., 2020; Markova et al., 2022).

In nanoparticle manufacture and quality analysis, generally, to maintain the colloidal stability, an electrical charge at the particle surface suspended in liquid (zeta potential value) of −30 to +30 mV is acceptable, owing to their optimal cationic or anionic, while nanoparticles with −10 to +10 mV are considered to have a near-neutral charge (Clogston and Patri, 2010). Meanwhile, further surface charge properties suitable for a biologically relevant delivery system require an in-depth and specific optimization.

In practice, given the negative charge of CNS endothelial cells, positively charged nanoparticles enable greater cellular uptake and BBB-penetrating activity compared to negatively charged nanoparticles. This is due to a favorable electrostatic attraction that induces tight junction opening, resulting in enhanced paracellular transport of ions, macromolecules, and hydrophilic drugs across cell barriers, and importantly, improves the BBB penetration of drug-loaded nanoparticles via adsorptive-mediated endocytosis (Roney et al., 2005; Del Amo et al., 2021). Intentional opening of the BBB presents considerable challenges, as it compromises a critical protective barrier of the central nervous system. Such disruption may induce neuroinflammatory responses, increase off-target permeability, and result in potential long-term CNS toxicity due to uncontrolled entry of exogenous substances and cumulative effects associated with repeated exposure. Accordingly, precise regulation of the extent and duration of BBB disruption, coupled with comprehensive safety evaluations, is essential. These risks must be carefully weighed against the anticipated therapeutic benefits to provide a balanced and realistic perspective for translational applications (Kochman et al., 2026).

**Figure 2:**
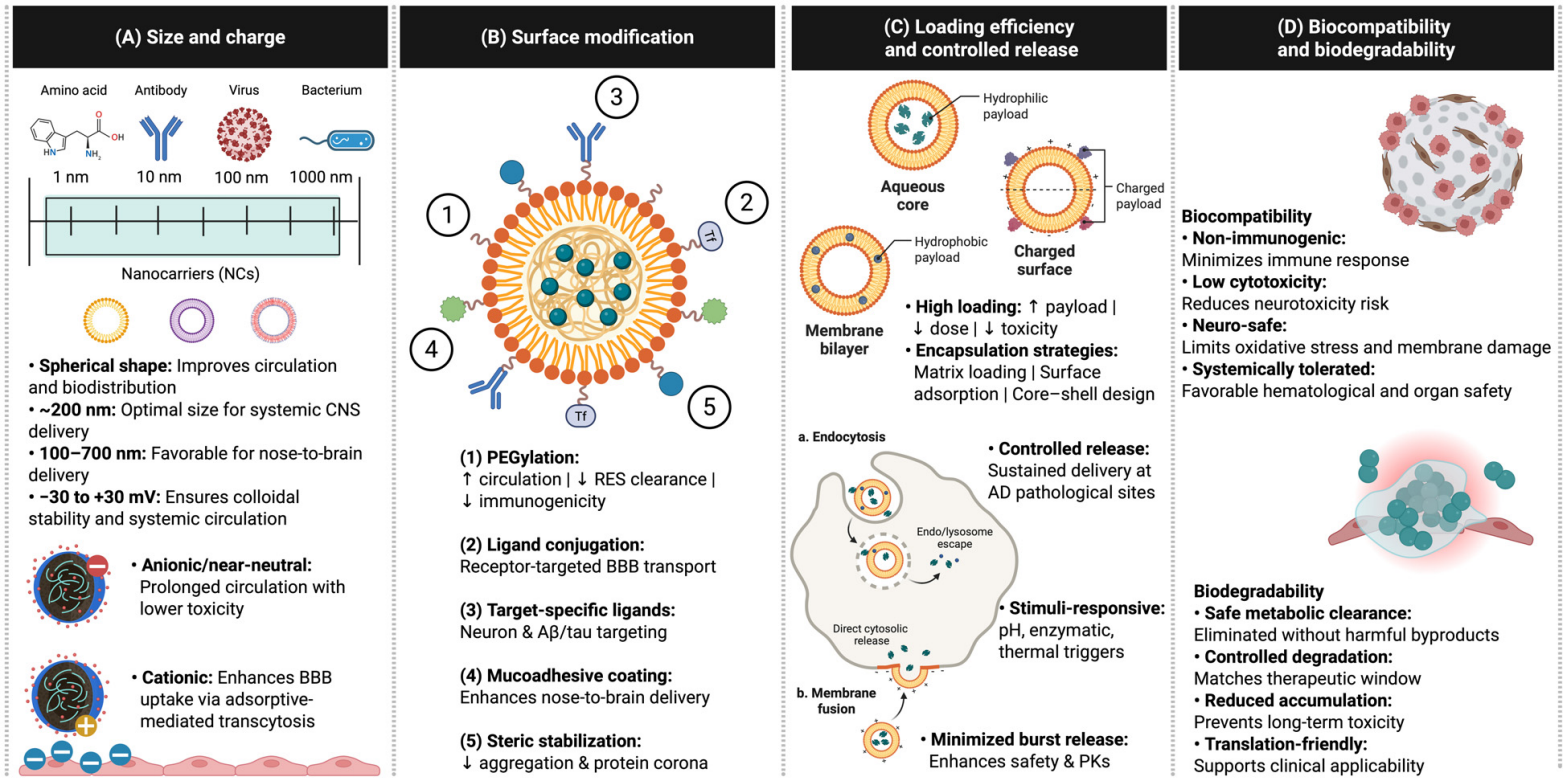
Design principles of nanoparticles for brain-targeted drug delivery in Alzheimer's disease. Schematic overview of key physicochemical and functional properties that influence nanoparticle performance for central nervous system delivery. **(A)** Size and surface charge critically regulate circulation, biodistribution, and BBB penetration, with nanoscale carriers supporting systemic and nose-to-brain transport. **(B)** Surface modification strategies, including PEGylation, ligand conjugation, target-specific ligands, mucoadhesive coatings, and steric stabilization for enhancing circulation time, receptor-mediated targeting, and formulation stability. **(C)** Efficient drug loading and controlled release enable sustained therapeutic delivery, intracellular trafficking, and stimuli-responsive payload release. **(D)** Biocompatibility and biodegradability are essential for minimizing immunogenicity, reducing neurotoxicity, supporting metabolic clearance, and improving clinical translatability. Figure created with BioRender.com and adapted from the “Liposome Based Drug Delivery” template by Jane He (BioRender). Licensed under CC BY 4.0 (https://creativecommons.org/licenses/by/4.0/).

In contrast, the low-negatively to neutral charged nanoparticles offer a lower toxicity risk and longer traveling time in the blood circulation (Gomes et al., 2025), which greatly benefits the passive targeting strategies in pathological conditions of Alzheimer's, where the BBB dysfunction arises (Alkhalifa et al., 2023; Lee and Funk, 2023) (Figure 2A). Examples of promising candidates with different surface charges include positively charged alendronate-loaded chitosan nanoparticles (zeta potential +23.8 mV), which have demonstrated enhanced brain delivery, leading to higher accumulation in the brains of intracerebroventricular-streptozotocin (ICV-STZ)-induced AD-like mice and improved pharmacokinetic profiles compared with free alendronate, suggesting more effective intranasal delivery (Zameer et al., 2021). Similarly, negatively charged memantine-loaded PEG-PLGA nanoparticles (zeta potential −22.4 mV) have shown excellent brain delivery, achieving sustained drug release following oral administration in APPswe/PS1dE9 (APP/PS1) and C57BL/6 mice (Sánchez-López et al., 2018). It is important to note that, in addition to particle size and charge, multiple factors critically influence a nanoparticle's ability to cross the BBB, including particle type, associated surface proteins, surface coatings, and the physiological state of the BBB (Hersh et al., 2022). Additionally, rather than relying solely on the mean zeta potential, surface charge heterogeneity should be considered, as subpopulations with distinct charges can markedly influence interactions with biological systems, including circulation, cellular uptake, and tissue distribution (Lee et al., 2011). Assessing the full zeta potential distribution can therefore provide a more accurate prediction of the nanoparticles' *in vivo* fate (Perry et al., 2016), and a charge variant isolation should be considered to minimize the unfavorable impacts *in vivo* (Khawli et al., 2010). Overall, the rational and tailored design should be carefully considered to achieve the desired performance and strategic delivery objectives.

#### 4.1.2 Surface modification

Surface engineering is recognized as an effective strategy for improving circulation time, physical stability, and even targeting selectivity and specificity. In a DDS, PEGylation is one of the versatile strategies developed for surface modification of nanoparticles. This chemical process involves a wide range of polyethylene glycol (PEG) conjugation chemistries and polymer structures, as different PEGylation chemistries and reaction conditions impact the therapeutic properties of drug delivery (Harris and Chess, 2003; Pfister and Morbidelli, 2014). Recent evidence suggests that PEGylation is generally employed to increase molecular weight and protect the payloads and/or drug nanoparticles from proteolytic degradation (Harris and Chess, 2003), thereby expanding their applicability in Alzheimer's and enabling specific designs that (i) prolong circulation time–as PEG chain can mitigate a rapid uptake by the RES (Kong et al., 2020), (ii) play a role in physical stability–by reducing particle agglomeration and/or protein corona effect (Guerrini et al., 2018) or providing a steric stability with targeted Aβ aggregates (Singh et al., 2022), (iii) improve BBB penetration (Du et al., 2024), (iv) reduce immunogenicity and toxicity (Kochman et al., 2026), and (v) enable controlled drug release and improved pharmacokinetics (Turjeman et al., 2015).

PEGylation helps extend the circulation time of nanoparticles through the enhanced permeability and retention (EPR) effect. However, it reduces cellular uptake and endosomal escape, leading to lower delivery efficiency. This challenge, known as the “PEG dilemma,” must be addressed for effective drug delivery. Several strategies have been addressed to overcome the PEG dilemma, including the use of targeting ligands, cleavable PEG systems, and peptides that enhance endosomal escape (Hatakeyama et al., 2011a,b). Taken together, PEGylation offers substantial advantages for nanoparticle stability and circulation; however, overcoming the PEG dilemma is crucial to ensure efficient cellular uptake and intracellular trafficking, ultimately maximizing the therapeutic effectiveness of nanoparticles.

Conjugation with the ligands that specifically interact with the targeted receptors, those on the BBB and neurons (e.g., folate, lactoferrin, transferrin, insulin, and LDL receptors), enhances the BBB penetration, neuron-targeting efficiency (Guo et al., 2020), and also increases drug bioavailability in the CNS (Gopalan et al., 2020; Guo et al., 2020; Sharma and Dang, 2023; Gomes et al., 2025). While ligand targeting of Alzheimer's pathology (e.g., hyperphosphorylated tau and Aβ plaques) is further incorporated as the ultimate objective, enabling specific delivery with triggered release of therapeutic agents at the pathological site (Pawar et al., 2025; Ross et al., 2018).

The intranasal therapeutic strategy has been increasingly explored as it facilitates the BBB bypass, granting a direct transportation of therapeutics into the brain via the nasal cavity; thus, the nanoparticles with mucoadhesivity are receiving considerable attention, as this formulation can overcome the physical and biological barriers of this approach–such as mucociliary clearance and poor nasal permeability (Sood et al., 2014; Dogra et al., 2024) (Figure 2B). Due to its excellent mucoadhesive properties, chitosan has emerged as a promising polymer for this approach, as it enhances drug adsorption via the intranasal route. However, interactions with mucus can induce polymer swelling, weaken mucoadhesive bond strength, and ultimately result in mucociliary clearance (Jiang et al., 2010). The cellulose derivatives like HPMC and synthetic polymers like polyacrylates (Carbopol/Carbomer) have also been widely recognized as polymers with mucoadhesive properties (Djekic, 2023). A literature on insulin gel formulated with Carbopol 934P and HPMC K4M showed sustained drug release *in vitro*, indicating its potential to provide prolonged therapeutic effects; however, its *in vivo* limitations remain and require further validation regarding long-term safety and repeated-dose effects (Porfiryeva et al., 2026).

#### 4.1.3 Loading efficiency and controlled release

Drug loading efficiency (also known as drug encapsulation efficiency) is used to evaluate the percentage of drug successfully encapsulated relative to the total drug amount, reflecting the nanoparticle's effectiveness in incorporating drugs. High drug loading efficiency is a critical requirement, as it offers economic efficiency—reducing drug loss during manufacturing—and enhances potential drug performance, by allowing lower doses to achieve the desired effect and thereby minimizing toxicity risk (Liu et al., 2020; Rasool et al., 2022). Typically, loading efficiency and loading capacity are two fundamental parameters that are inversely related in drug delivery systems. In practice, increasing one often leads to a decrease in the other; for example, increasing the therapeutic-to-matrix ratio—thereby raising the initial drug input and potential loading—often results in a marked reduction in encapsulation efficiency (Xu and Hanna, 2006). This trade-off arises from the finite capacity of carriers, saturation effects, and the nature of drug–carrier interactions (Xu and Hanna, 2006; Palanikumar et al., 2018). Thus, formulation design typically aims to balance these two parameters to achieve both efficient drug utilization and a therapeutically effective payload.

Principally, to achieve the desired loading efficiency, many key factors are documented; for example, (i) particle size and surface-area-to-volume ratio, where reducing the sphere size increases the surface area per unit volume, resulting in higher drug loading efficiency (Dawes et al., 2009; De et al., 2022); (ii) porosity, including pore structure and volume, is a critical factor for porous nanoparticles, as a larger pore volume combined with a larger surface area enhances a drug loading (Manaia et al., 2017); (iii) biomaterial properties (e.g., structure, composition, molecular weight, functional groups, and hydrophobicity), which is suggested to influence a solid-state drug-polymer solubility, in which higher solid-state drug solubility lead to higher drug entrapment (Panyam et al., 2004; Wang et al., 2019); (iv) drug hydrophobicity and miscibility with nanoparticles, a key factor that indicates the strength of drug-polymer interactions, where greater interactions are more likely enables higher drug loading. Furthermore, alteration in nanoparticles' physicochemical properties (e.g., structure, density, and size) can also be achieved through a drug loading process (Jäger et al., 2018); and (v) manufacturing process—such as surface loading and matrix loading system—offers distinct mechanisms of action in drug encapsulation that vary the drug loading efficiency (Wang et al., 2019).

Through controlled drug release, nanoparticles offer several advantageous features for the treatment of AD, where precise delivery of the drug to the pathological site is required. Ideally, nanoparticles with controlled release function should be able to preserve the drug within their structure. This facilitates both reducing drug loss and preventing the drug from degradation during transportation, and slowly releases the drug at the targeted pathological site. This, in turn, enhances the drug efficacy while reducing the risk of systemic toxicity, as it helps prevent rapid burst release and minimizes the need for repeated dosing (Natarajan et al., 2014; Adepu and Ramakrishna, 2021; Askarizadeh et al., 2023). Enzyme-responsive self-assembled nanovesicles have shown considerable potential for delivering water-soluble drugs in AD therapy. These vesicles respond specifically to the brain microenvironment, where the endogenous legumain endopeptidase cleaves a target peptide, triggering cross-linking and the formation of micrometer-scale vesicles, which in turn reduces efflux across the BBB in SAMP8 mice. *In vitro* studies further demonstrate that the cross-linked vesicles act as a reservoir, sustaining cumulative drug release over 48 h under intracellular-mimicking conditions. Despite these promising results, the limited understanding of *in vivo* controlled drug release presents a critical challenge, potentially hindering the clinical translation of these stimuli-responsive nanovesicles (Jiang et al., 2021) (Figure 2C).

#### 4.1.4 Biodegradability and biocompatibility

The choice of biomaterial is a crucial step in nanoparticle development, as the properties of biomaterials reflect not only physical characteristics but also their biodegradation and biocompatibility profiles, in which the suitable biomaterials should exhibit non-immunogenicity (Nunes et al., 2022; Gao et al., 2026). Recently, several biodegradable and biocompatible biomaterials [e.g., lipid (Olbrich et al., 2002), chitosan (Bastiaens et al., 2019), protein-based (Li and Li, 2014; Narayan et al., 2025)] have attracted considerable attention in the nanoparticle arena; however, they share the same goal of facilitating ideal characteristics and ensuring safety, including (i) being readily metabolized and eliminated from the body after accomplishing their desired biological functions, without cytotoxicity-derived byproducts, and (ii) possessing a suitable half-life that is compatible with the therapeutic period of targeted disease (Li and Li, 2014).

Advanced DDS with precise targeting can lead to the accumulation of nanoparticles at the target site, where undesired interactions can occur, resulting in neurotoxicity mediated by oxidative stress, disruption of membrane integrity, and DNA damage (Gao et al., 2026); thereby, the neuroprotective nanoparticles have been strongly emphasized, presenting significant opportunities in Alzheimer's application (Zhang et al., 2021; López et al., 2020). While various clinically relevant nanoparticles contain non-degradable inorganic components, their long-term biological applications raise important concerns regarding clearance mechanisms. The potential for long-term accumulation of magnetic nanoparticles has been highlighted, with a significant decrease observed in the liver and complete absence in the spleen after 60 days; however, extensive preclinical studies are still needed due to the uncertain effects of their aggregation in these organs (Soares et al., 2022). Thus, ensuring biodegradability is essential to minimize long-term retention in the body and to enhance the safety and clinical viability of these nanoparticle systems. Systemic toxicity evaluations—such as hematological parameters, biochemical profile, and organ histopathology—are also emphasized, as abnormality of these can occur via intravenous injection, a standard route of administration, for drug nanoparticles in the treatment of AD (Ma et al., 2021; Saini et al., 2021; Gong et al., 2024).

Collectively, the physicochemical-optimized nanoparticles can be designed as a highly promising platform for effective anti-Alzheimer's drug delivery, in which, within this criteria window, nanoparticles exhibit (i) prolong systemic circulation time, reducing rapid clearance by immune system and enhance passive targeting due to the pathology of AD (Sánchez-López et al., 2018; Gajbhiye et al., 2020; Kochman et al., 2026), (ii) enhance interaction with brain endothelial cells and overcome the BBB for systemic delivery pathway (Guo et al., 2020), while circumvent the mucociliary clearance and enhance nasal permeability for nose-to-brain delivery pathway (Sood et al., 2014; Dogra et al., 2024), (iii) facilitate high drug loading and controlled release (Natarajan et al., 2014; Liu et al., 2020; Adepu and Ramakrishna, 2021; Rasool et al., 2022; Askarizadeh et al., 2023), and (iv) achieve biosafety as biocompatible nanoparticles in Alzheimer's application (Gao et al., 2026) (Figure 2D).

### 4.2 Nanoparticle platforms: fabrication, properties, and translational challenges

#### 4.2.1 Lipid-based nanoparticles (LBNs)

##### 4.2.1.1 Solid lipid nanoparticles (SLNs)

**Figure 3:**
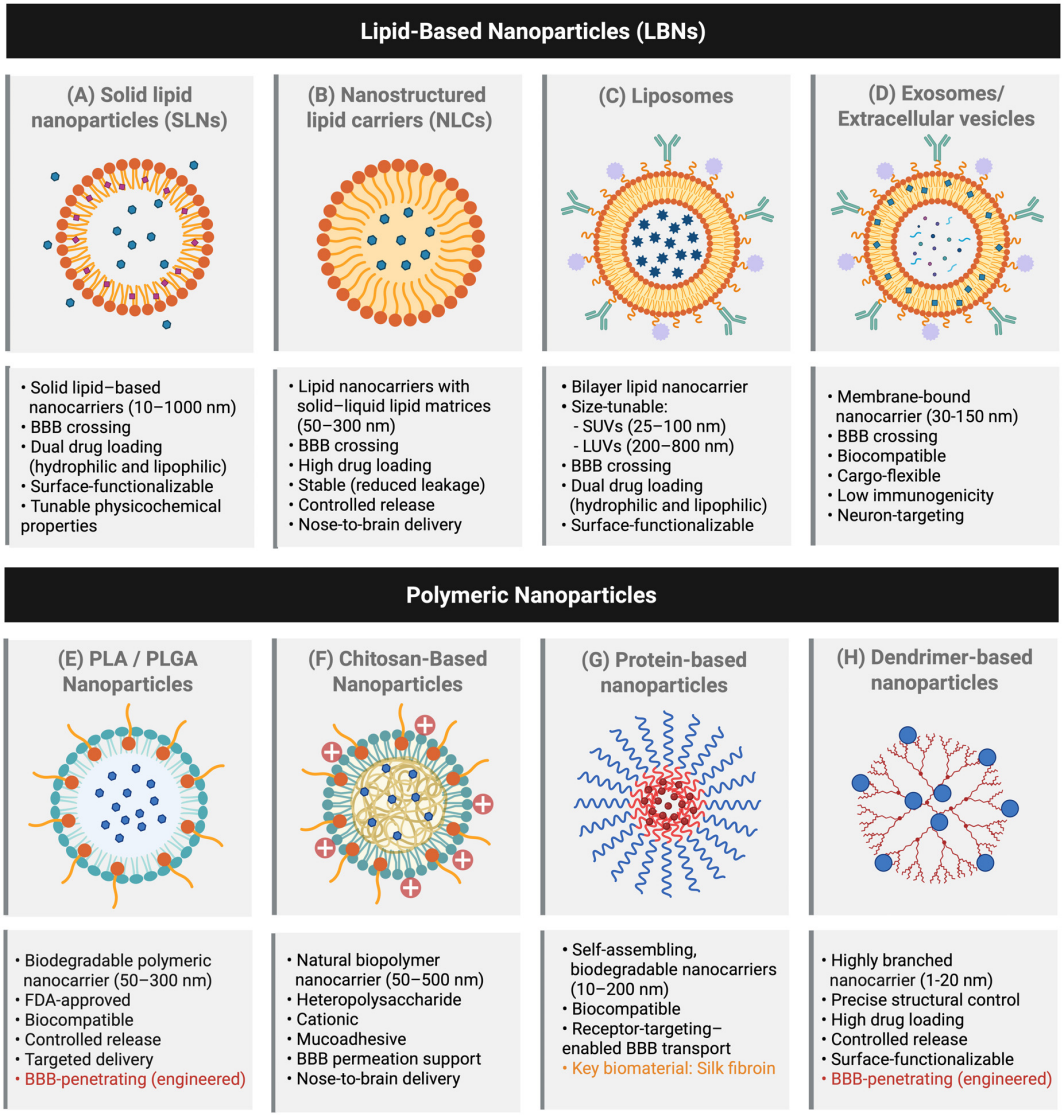
Representative nanoparticle platforms for brain-targeted drug delivery in Alzheimer's disease. Overview of major nanoparticle systems investigated for enhancing blood–brain barrier penetration and therapeutic delivery in Alzheimer's disease. Lipid-based nanoparticles (LBNs) include **(A)** solid lipid nanoparticles (SLNs), which provide stable lipid matrices for dual drug loading; **(B)** nanostructured lipid carriers (NLCs), offering improved drug incorporation and controlled release; **(C)** liposomes, bilayer vesicles capable of encapsulating both hydrophilic and lipophilic agents; and **(D)** exosomes or extracellular vesicles, naturally derived carriers with inherent biocompatibility and neuron-targeting potential. Polymeric nanoparticles comprise **(E)** PLA/PLGA-based carriers with tunable biodegradability and controlled release properties; **(F)** chitosan-based nanoparticles that enhance mucoadhesion and support nose-to-brain transport; **(G)** protein-based nanoparticles, including silk fibroin systems, which enable receptor-targeted delivery; and **(H)** dendrimer-based nanoparticles characterized by highly branched architectures that support high drug loading and surface functionalization. Figure created with BioRender.com and adapted from the “Icon Pack—Drug Delivery Vesicles” template by Martina Maritan (BioRender). Licensed under CC BY 4.0 (https://creativecommons.org/licenses/by/4.0/).

Solid lipid nanoparticles (SLNs), which are unilamellar lipid-based nanoparticles, constitute a well-established and highly versatile class of lipid nanoparticles owing to their solid hydrophobic lipid core, which effectively accommodates both hydrophobic and hydrophilic therapeutic agents (Satapathy et al., 2021). SLNs can be reliably fabricated using a range of scalable and biocompatible techniques, including high-shear homogenization (Saini et al., 2021; Shivananjegowda et al., 2023), solvent emulsification (Yusuf et al., 2013; Khishvand et al., 2024), solvent injection (Yasir et al., 2018), and microfluidic methods (Bian et al., 2024). Careful optimization of formulation and processing parameters enables robust and reproducible control over critical physicochemical attributes, including particle size, surface charge, stability, drug encapsulation efficiency, and release kinetics (Üner, 2006). These preparation strategies enable the fabrication of SLNs over a broad size range of approximately 10–1,000 nm, allowing flexibility in size tuning to meet the requirements for anti-Alzheimer's drug delivery (Satapathy et al., 2021) (Figure 3A).

To date, beyond nanoparticles designed solely based on physicochemical properties, extensive optimization efforts have produced several promising functionalized SLN formulations for the treatment of AD. Among these, vinpocetine-derived ionizable-lipidoid nanoparticles (VIP) have emerged as an interesting, promising brain-targeting platform model for gene delivery, addressing not only the challenge of crossing the BBB but also the critical issue of microcirculation disorders. These disorders, which are common in many brain diseases, for example, a progression of mild cognitive impairment to AD, lead to a severe reduction in cerebral blood flow (up to 40%), can significantly limit drug accumulation within brain lesions even after BBB penetration. Incorporation of cerebral blood flow-regulatory compounds—such as vinpocetine—into an ionizable lipid demonstrates outstanding potential, highlighting a brain-protective activity and safe administration in APP/PS1 mice (Bian et al., 2024). According to Bian et al., VIP is proposed to enhance drug accumulation in the brain by improving cerebral blood flow, even in regions affected by microvascular blockage and ischemia, as observed in APP/PS1 mice, cerebral gliomas, and fungal meningitis models. By restoring local perfusion at lesion sites, VIP facilitates increased drug delivery. This mechanism is further supported by evidence that pharmacologic modulation of cerebral blood flow can positively influence BBB function under pathological conditions, indicating that VIP-based strategies may improve both vascular perfusion and therapeutic efficacy through multiple complementary pathways.

##### 4.2.1.2 Nanostructured lipid carriers (NLCs)

Nanostructured lipid carriers (NLCs) are second-generation lipid carriers that share similar physicochemical properties with SLNs. Although SLNs and NLCs have proven to be potential candidates for the treatment of AD, as evidenced by their effectiveness in both hydrophilic and hydrophobic therapeutic agent transportation across the BBB (Amiri et al., 2021; Dighe et al., 2023), NLCs have more recently emerged as the dominant platforms for an alternative intranasal administration—nose-to-brain delivery (Shehata et al., 2023b,a; Ali et al., 2024; Tekade et al., 2024; Khan et al., 2025; Samala et al., 2025). One possible reason for this popularity is the structure of NLCs, which is well-suited for drug delivery. NLCs possess a solid lipid matrix containing nano-oil compartments, resulting in higher drug-loading efficiency, reduced drug leakage during storage, and provide a greater control over drug release (Duan et al., 2020; Witika et al., 2022; Ali et al., 2024), while the presence of liquid lipids prevents crystallinity and polymorphic transitions, thereby ensuring long-term stability (Ali et al., 2024) (Figure 3B). While drug expulsion from SLNs is caused by a reduction of amorphous regions in the lipid matrix due to a polymorphic transition from the less-ordered α-form to the more-ordered β-form, which reduces the space available for drug molecules, the drug is consequently expelled from the lipid core and may migrate to the particle surface or surrounding medium, leading to a gradual decrease in drug loading efficiency over time (Westesen et al., 1997; Westesen, 2000). Additionally, NLCs demonstrated superior drug entrapment compared to SLNs, as shown by the quantitative measurement of hydrochlorothiazide loading, with NLCs achieving approximately 90% and SLNs around 80%. This superior performance of NLCs over SLNs was evident in terms of drug entrapment efficiency (Mura et al., 2021).

NLCs significantly improved quercetin's major limitation, its solubility, and enhanced *in vitro* cellular uptake and BBB penetration. In the Wistar rat model, the quercetin-loaded NLCs demonstrated a great potential in ameliorating the oxidation and inflammation, along with a reduction of Aβ accumulation. This study also highlighted a promise of NLCs in the treatment application of AD via an intranasal route, as evidenced by increased accumulation and prolonged retention time of quercetin-loaded NLCs in the nasal cavity (~8 h) (Satpathy et al., 2025).

##### 4.2.1.3 Liposomes

Generally, liposomes are spherical organic nanoparticles with a bilayer lipid structure–whose conventional compositions are mainly characterized as phospholipid and lipids, such as monosialoganglioside, phosphatidylcholines, sphingomyelin, and 1,2-distearoyl-sn-glycero-3-phosphatidyl choline, offering an interior aqueous core and exterior impermeable lipophilic phospholipid layer. With this characteristic, liposomes exhibit an excellent protective effect for the drug, as the aqueous phase stabilizes the polar segment, along with shielding the nonpolar segment of the drug. Additionally, bilayer lipid structure facilitates the transport of lipid soluble drug across the cell membrane (Lichtenberg and Barenholz, 1988; Hernandez and Shukla, 2022).

Owing to method preparation, liposomes can be classified into three categories: (i) multilamellar vesicles (MLVs) are heterogeneous-sized liposomes (500–5,000 nm) fabricated by simultaneous formation and gentle shaking. With optimized homogenizations, (ii) small unilamellar vesicles (SUVs; 25–100 nm) and (iii) large unilamellar vesicles (LUVs; 200–800 nm), both are unilayer-structured liposomes, can be synthesized (Spuch and Navarro, 2011). The pH, temperature, and cholesterol content have been recognized as key factors influencing the physicochemical stability of liposomes (Sułkowski et al., 2005). Among these, cholesterol has a particularly strong effect on the electrical permeability and stability of phosphatidylcholine liposomes, suggesting that it modulates the hydrophobic barrier of the bilayer core and thereby influences membrane permeability and overall stability (Raffy and Teissié, 1999). Due to its ability to regulate membrane fluidity and stability, cholesterol is considered a crucial component in liposome formulation, serving as an important modulator of membrane permeability, elasticity, and stiffness in eukaryotic cell membranes, typically at 30–50 mol % of the total lipid fraction (Jovanović et al., 2018). Thus, optimizing liposome composition, particularly cholesterol content, can enable the rational design of liposomes with improved drug retention and enhanced resistance to *in vivo* degradation, ultimately facilitating their clinical translation (Figure 3C).

Functionalized liposomes have been extensively studied for targeting Alzheimer's pathological conditions owing to their exploitable physicochemical properties, which can be optimized—particularly through flexible and broad surface-engineering strategies; for example, promoting the BBB penetration with enhanced cellular uptake in brain endothelial cells using a cell-penetrating peptides (Arora et al., 2020; Muolokwu et al., 2024) or ligands (Mittal et al., 2020), enabling brain-targeting for neurons (Kuo and Tsao, 2017; Mittal et al., 2020), astrocytes (Kuo and Lin, 2015; Gomes et al., 2025), and microglia (Wei et al., 2025), as well as supporting receptor-mediated transcytosis approach (Arora et al., 2020). Among the surface modifications targeting AD, as mentioned above, Apolipoprotein E (ApoE)-modified liposomes demonstrated success in the enhancement of *in vitro* cellular uptake in bEnd.3 and N2a cell lines and transportation across the BBB. Encapsulation of resveratrol and salidroside in liposomes improved therapeutic efficacy in APP/PS-1 mice, ultimately reducing their learning and memory impairment (Chen M.-H. et al., 2023).

##### 4.2.1.4 Exosomes/extracellular vesicles

Extracellular vesicles (EVs) are membrane-bound particles that contain various biomolecules (e.g., proteins, lipids, and nucleic acids) and are produced by all cell types and released into the extracellular space to perform specific cellular functions. Exosomes (30–150 nm) are a subtype of EVs that originate from the endosomal pathway in cells and are secreted into biological fluids, including plasma, urine, saliva, and cerebrospinal fluid (Ebadpour et al., 2025; Jang et al., 2025). Typically, exosomes have been extensively used as biomarkers and have proven beneficial in medical diagnostics for AD (Aharon et al., 2020). More recently, the medical application of exosomes has expanded to DDS, particularly involving neuron-derived (Ebadpour et al., 2025), stem cell-derived (Ebadpour et al., 2025), and brain endothelial-derived exosomes (Pan et al., 2020). This expansion is attributed to several favorable properties of exosome-based systems, including (i) high biocompatibility, as exosomal membrane structures retain certain characteristics of their parent cells (Pan et al., 2020); (ii) the potential to facilitate BBB transport via transcytosis under specific conditions (Saint-Pol et al., 2020); and (iii) the capacity to load therapeutic agents and enhance therapeutic efficacy (Yin et al., 2023) (Figure 3D).

A key consideration of exosomes is that the challenges in extracting biological fluid-derived exosomes, for example, in plasma, where enrichment of biological molecules and various exosome species exists, thereby an issue like the corona effect occurs (Voke et al., 2025). The fluid complexity leads to several contamination issues, as the standard and efficient preparation methods, apart from the conventional methods (e.g., ultracentrifugation, ultrafiltration, gel filtration technique, etc.), remain underexplored, thus restricting the clinical development of exosome-based therapies (Yakubovich et al., 2022). Beyond purification, EVs characterization procedures that ensure transparency, reproducibility, and comparability of EV studies across laboratories remain challenging. The MISEV2018 guidelines offer standardized recommendations to improve the rigor and reproducibility of EV studies, which include (i) considerations for EV separation/enrichment, (ii) EV characterization (e.g., size, concentration, morphology, molecular marker, protein content-based EVs, etc.), (iii) EV-associated and EV-excluded biological activities, and (iv) reporting standards–reporting the EV source, isolation, characterization, and experimental design (Théry et al., 2018).

The use of exosomes in AD applications is gradually increasing. For instance, human umbilical cord mesenchymal stem cell (hUC-MSC)-derived exosomes have been engineered with a bioinspired neural cell-targeting peptide, rabies virus glycoprotein 29 (RVG29), to facilitate targeted delivery to microglial and neuronal cells. Encapsulation of BACE1 siRNA and berberine into these engineered exosomes enhanced cognitive function and nerve repair in 5 × FAD mice following intranasal administration (Sun et al., 2025).

#### 4.2.2 Polymeric nanoparticles

##### 4.2.2.1 Poly(lactic-co-glycolic acid)-based nanoparticles

Poly(lactic acid) (PLA) is widely used as an important biomaterial in DDS due to their excellent biocompatibility with the human body and undergoes degradation and clearance processes in which their by-products are non-toxic (Kumar and Kumar, 2019). PLA is a hydrophobic aliphatic biopolymer–derived from the lactic fermentation process–and is composed of poly(l-lactic acid), poly(d-lactic acid), poly(d,l-lactic acid), and meso-PLA. PLA contains a chiral α-carbon, which is commonly described using the classical d- and l-form, or alternatively as the *R*- and *S*-configurations. Accordingly, PLA exists in enantiomeric forms, namely poly(d-lactic acid) (PDLA) and poly(l-lactic acid) (PLLA).

Generally, PLA is extensively blended with other biopolymers; the most widely used polymer is poly(glycolic acid) (PGA), resulting in a combined polyester biopolymer named poly(lactic-co-glycolic acid) (PLGA) (Kumar and Kumar, 2019). PLGA typically refers to poly(d,l-lactic-co-glycolic acid), in which the d- and l-forms of lactic acid are present in equal proportions (Makadia and Siegel, 2011). Variations in the molar ratio of lactic acid to glycolic acid in the polymer chain influence the copolymer's properties, such as hydrophilicity, molecular weight, and degree of crystallinity, thereby governing its degradation rate and drug release kinetics. As reported by Makadia and Siegel, a 50:50 PLA/PGA ratio exhibits the fastest degradation.

PLGA has been approved by the U.S. Food and Drug Administration (FDA) and the European Medicines Agency (EMA) and has attracted considerable attention for drug nanoparticle development (Kumar and Kumar, 2019; Zeb et al., 2022). Given the distinctive characteristics of PLA and PGA, functionalized PLGA nanoparticles can be favorably tailored by adjusting the PLA/PGA ratio, which in turn influences their overall hierarchical structure and physical stability as well as biodegradability (Cui et al., 2025), drug loading, and controlled release (Lim et al., 2022) (Figure 3E). Curcumin-loaded PLGA nanoparticles exhibited excellent biocompatibility, neuroprotective effects, and improved learning and memory ability in APP/PS1 mice (Zeng et al., 2023). PLGA nanoparticles incorporating mefenamic acid facilitated intranasal delivery for AD treatment due to their high mucoadhesive activity. This formulation provided higher drug adsorption via the intranasal route compared with a free drug suspension (Dogra et al., 2025). Despite their potential as targeted PLGA-based DDS, PLGA nanoparticles still face several challenges that hinder the translation of research findings into clinical applications. These challenges include: (i) manufacturing issues, such as low drug-loading capacity, high production costs, and scale-up limitations in manufacturing (Na et al., 2023); and (ii) delivery-related issues, as PLGA nanoparticles require further engineering to achieve high selectivity and specificity, overcome BBB restrictions, and effectively target pathological sites, and/or multi-targeting strategies may be necessary as AD exhibits multiple pathological targets (Lu et al., 2023).

##### 4.2.2.2 Chitosan-based nanoparticles

Chitosan is a natural biopolymer with a well-defined structure as a linear binary heteropolysaccharide. It is derived from the alkaline *N*-deacetylation of chitin, which is typically found in several organisms, particularly fungi and crustaceans (Bastiaens et al., 2019). This process is commonly carried out using concentrated sodium hydroxide under heated conditions for an extended period of time (Abd Elgadir et al., 2015). Chitosan has been the focus of extensive attention in broad-spectrum applications in biomedical and pharmaceutical fields due to its favorable properties that satisfy the material requirements for DDS, including biocompatibility, biodegradability, and importantly, its outstanding cationic property–which additionally offers a significant potential in anionic DDS, mucoadhesion, controlled drug release, and permeability characteristic (Abd Elgadir et al., 2015). Multiple drug delivery strategies derived from chitosan's cationic property have expanded its applications, including oral, transmucosal, pulmonary, and transdermal nanoparticles (Iacob et al., 2021) (Figure 3F).

Owing to its outstanding mucoadhesive activity, chitosan has demonstrated significant potential in the treatment of AD (Elnaggar et al., 2015). Chitosan-engineered surface nanoparticles have been developed, facilitating enhanced nasal adsorption, ultimately, enhanced brain delivery to Alzheimer's pathological sites (Gothwal et al., 2023). A peracetylated chitosan oligosaccharides (PACOs) demonstrated a promising potential in AD treatment in Sprague–Dawley rats, enabling a neuroprotective effect against Aβ-induced cognitive deficits. This study also highlighted the improvement of cognitive function-derived PACOs, including enhanced learning and memory function, attenuated nerve damage repair, and inhibited neuronal apoptosis through the PI3K/Akt/GSK3β signaling pathway (Hao et al., 2023).

Nevertheless, the worldwide challenges of chitosan in drug nanoparticle technology remain, such as the limitation of the material source, time-consuming manufacture, unreliable production yield, and non-environmentally friendly synthesis due to the requirement of harsh chemical treatment (Alemu et al., 2023). Thus, chitosan's standard processing procedure should be further considered sustainable and suitable for industrial-scale application (Kou et al., 2021).

##### 4.2.2.3 Protein-based nanoparticles

For a decade, proteins have emerged as a crucial biomaterial in DDS, as most proteins exhibit a self-assembling characteristic, resulting from both inter- and intra-molecular interactions, such as hydrophobic, electrostatic, hydrogen bonding, and van der Waals, that form between protein chains and/or fragments. This behavior is advantageous for nanoparticle formation, leading to the assembly of protein complexes with a predominantly hydrophobic inner core, while hydrophilic residues are more likely to be presented on the particle surface, facilitating interactions with surrounding water/solvent molecules (Estrada and Champion, 2015) (Figure 3G).

In recent years of Alzheimer's drug delivery research, various proteins have emerged as potential candidates due to their distinctive properties and tailored design strategies. In mice model. serum albumin-based nanoparticle enhanced the *in vivo* brain-to-plasma concentration ratio of *R*-flurbiprofen compared with intranasal administration of *R*-flurbiprofen alone. A significant improvement in basal and maximal mitochondrial respiration *in vitro* was also observed, suggesting a potential approach for treating AD-related mitochondrial dysfunction via intranasal delivery (Wong and Ho, 2018). Zein/lactoferrin composite nanoparticles improved the pharmacokinetic profile of the TrkB agonist by increasing plasma *C*_max_ and half-life (*t*_1/2_), ultimately ameliorating cognitive dysfunction in mice (Wang et al., 2023b). H-ferritin nanoparticles have emerged as a therapeutic nanoplatform for inflammation-related AD, exhibiting favorable properties that enhance curcumin encapsulation and neuroprotective effects by reducing microgliosis and astrogliosis, while also improving cognitive performance in 5xFAD mice (Morasso et al., 2024); additionally, improving the stability and solubility of Bisdemethoxycurcumin (BDC)-loaded nanoformulation (Gagliardi et al., 2022). Transferrin (TfR) nanoparticles were manufactured to encapsulate Donepezil and were surface-modified with cRGD-conjugated chitosan, as an improved model of a dual-brain targeting DDS. This strategy ideally enables receptor-mediated transcytosis (RMT) across the BBB via TfR functionality, while cRGD targets integrins overexpressed on the inflamed BBB and neurovascular units proximal to Aβ aggregates; together, these mechanisms enhance brain delivery and cellular uptake at specific AD pathological (Wang et al., 2025a) Casein micelles fabricated via self-assembly demonstrated strong potential for loading poorly soluble drugs, along with high biocompatibility and biodegradability. Piperine-loaded casein micelles exhibited therapeutic potential for AD, as evidenced by enhanced neuroprotective effects, including antioxidant activity, restoration of mitochondrial homeostasis, and protection against Aβ(_1 − 42_)-induced cytotoxicity and apoptosis (Mishra et al., 2026).

Silk fibroin has emerged as a promising biomaterial in nanoparticles due to its versatile functionality–high mechanical strength, flexibility, low immunogenicity, biodegradability, and biocompatibility (Bhattacharya et al., 2022)–and adaptability in various formats—including fibers (Xiaodan et al., 2016), films (Ma et al., 2025), hydrogels (Kaewchuchuen et al., 2024), powders (Rajkhowa et al., 2012), and micro- or nanoparticles (Roamcharern et al., 2025)—enabling broad applications in manufacturing and rational drug delivery design. The biosafety of silk has been recognized by several organizations, including the FDA, which has allowed drug and device manufacturers to register silk for clinical trials since 2007 (Holland et al., 2019). This recognition has facilitated extensive research highlighting its broad biomedical applications, ranging from medical sutures (Muffly et al., 2011) to nanoparticle-based drug delivery platforms (Roamcharern et al., 2025).

In the context of AD and other neurodegenerative disorders, silk nanoparticles enable versatile therapeutic delivery, including drugs and other active agents targeting specific cell types (Mathur and Gupta, 2010) and have demonstrated the ability to bypass the BBB and reach deep brain regions (Yonesi et al., 2021). Furthermore, silk fibroin itself exhibits therapeutic effects, such as attenuating spatial learning and memory impairments and preventing neuronal damage in Aβ_25 − 35_-induced rat models through the reduction of Tau hyperphosphorylation and suppression of neuroinflammation (Yao et al., 2022), and preventing Aβ- and/or reactive oxygen species-induced inflammation and apoptosis, along with improved cognitive function by increasing acetylcholinesterase (De Giorgio et al., 2024).

##### 4.2.2.4 Dendrimer-based nanoparticles

Dendrimers are highly branched macromolecules that consist of a bi- or multifunctional core from which branching units radiate outward. Their structure is organized in generations branching toward the particle surface; thereby, a higher generation results in a greater number of functional groups at the particle surface and a higher packed surface. These characteristics of dendrimers allow a rational design for drug delivery, particularly a well-defined branched architecture with precise control over molecular shape, size, and terminal functional groups. This should provide a compatible chemical environment in the particle core for the therapeutics, enhancing their loading capacity while also offering a shielding/protective function (Moreira et al., 2023; Pérez-Carrión and Posadas, 2023) (Figure 3H). Oleic acid-conjugated PAMAM G4 was synthesized as a model for the delivery of donepezil via the intranasal route in Sprague–Dawley (SD) rats, proposing to bypass the BBB and minimize systemic toxicity. This research strengthens the advantages derived from dendrimers, such as enhanced structural adaptability, stability, and mucosal penetration, as well as improved drug loading efficiency and sustained drug release (Suthar et al., 2025).

#### 4.2.3 Antibody-drug conjugates (ADCs)

Antibody-drug conjugates (ADCs) are engineered antibodies linked to therapeutic agents, designed to minimize off-target effects through the antibody's high selectivity to the target and BBB-penetrating activity while providing therapeutic activity via the attached agents. Many antibody engineering approaches have emerged as novel technologies for the treatment of AD, offering diverse and valuable functions (Punyakoti et al., 2023; Amelimojarad and Amelimojarad, 2025). Promising examples of ADCs, such as PROTAC-antibody conjugates—classified as antibody-mediated targeted protein degraders—enable the degradation of Tau and Aβ, critical targets in AD, while providing cell selectivity and enhanced membrane permeability due to the inherent advantages of antibodies (Feng and Wang, 2025). Similarly, Morphomer^®^ ADC (morADC), which combines monoclonal antibodies with brain-penetrating small molecules, provides selective targeting of toxic proteins in AD (Kim et al., 2025). Although ADCs ideally enable non-invasive transport mechanisms with high specificity and biocompatibility for crossing the BBB, several challenges remain. These include low stability and short half-life—due to susceptibility to proteolytic degradation and rapid renal clearance (Zhou et al., 2021)—poor drug efficacy (Gu et al., 2024), and costly manufacturing processes (Yang, 2025).

#### 4.2.4 Stimuli-responsive nanoparticles

Factors influencing drug release are incorporated into the nanoparticle, enabling distinct controlled-release mechanisms and paving the way for various optimization and design strategies. These include diffusion-controlled release, solvent-controlled release (e.g., osmosis- and swelling-controlled release), degradation-controlled release, and stimuli-responsive release (e.g., pH, ionic strength, temperature, and electric or magnetic fields) (Lee and Yeo, 2015). The nanoparticles with stimuli-responsive function targeted for Alzheimer's treatment will be further discussed in the following section to achieve an in-depth understanding of their concept and pharmaceutical application for AD.

##### 4.2.4.1 pH-responsive nanoparticles

The classical strategy of pH-responsive nanoparticles in drug delivery—particularly lipid-based and polymeric nanoparticles—enables controlled drug release at specific pH conditions relevant to the targeted pathological lesions. This release can occur through different mechanisms, such as nanoparticle dissolution—an instability that arises under specific pH conditions, typically at lower pH, leading to matrix dissolution—and polymer swelling, a process in which nanoparticles swell at certain pH conditions, both ultimately resulting in drug release (Karimi et al., 2016). This has inspired the use of pH-responsive elements to improve drug delivery to the brain; for instance, a nanoparticle system incorporating an acid-cleavable polyethylene glycol (PEG) linker for the T7 peptide—which selectively binds to TfR receptors on the BBB—allows the acid-sensitive T7 to be cleaved from the nanoparticles within endo/lysosomes, supporting enhanced cellular uptake, rapid endo/lysosomal escape, and efficient transcytosis, ultimately improving *in vivo* distribution in AD mice (Cai et al., 2020).

##### 4.2.4.2 ROS-responsive nanoparticles

The increased oxidative damage in the CNS in Alzheimer's patients results from an imbalance of cellular oxidation, owing to an accumulation of reactive oxygen species (ROS). Several Alzheimer's pathological conditions are reported to be closely interrelated to oxidative stress; thus, ROS emerges as a potent stimulus for targeting AD (Yu and Luo, 2024). The FTBR nanoparticles, which incorporate a ROS-responsive linker, demonstrate responsiveness under elevated ROS conditions, facilitating therapeutic release at ROS-related pathological brain sites in AD mice following intranasal administration (Chen et al., 2025). The multifunctional nanoparticle has created to inhibit Aβ aggregation, along with an elimination of ROS, such as the curcumin-loaded cerium oxide nanoparticles, demonstrating excellent therapeutic effects by reducing Aβ accumulation, reducing oxidative stress, and improving cognitive ability in mice (Wang et al., 2024).

##### 4.2.4.3 Temperature-responsive nanoparticles

In DDS, nanoparticles can be engineered to exhibit temperature-responsive characteristics, including temperature-induced phase transitions, conformational changes, drug release, and structural transformations (Dai et al., 2025). Because temperature critically influences the physicochemical properties of biomaterials used as nanoparticles, rationally designed temperature-responsive systems can enable precise targeting and controlled drug release. In AD, the BBB represents a major limitation, hindering the delivery of nanoparticles to specific pathological sites. Through rational design based on this approach, a flower-shaped hollow nano-ruthenium carrier exhibits strong BBB-penetrating capability triggered by a photothermal effect *in vitro*, arising from the temperature-stimulated phase change of tetradecyl alcohol, which acts as a thermal response switch. This design additionally enables prolonged circulation time and controlled release of the therapeutic agent, ultimately achieving precise delivery to tau-related Alzheimer's pathological lesions in AD mice (Zhou et al., 2020).

Versatile temperature-responsive nanoparticles have also been designed to target Aβ aggregates. One example is polymer-dispersed liquid crystal (PDLC) nanoparticles, which exhibit photothermal sensitivity with a strong near-infrared absorption, enabling temperature-induced phase transitions. Upon incorporation of an Aβ-specific ligand, these nanoparticles display high affinity for Aβ aggregates and allow photothermal-triggered controlled release of the therapeutic agent, ultimately enhancing anti-amyloid activity through a combined chemo-photothermal therapeutic approach (Lai et al., 2020).

##### 4.2.4.4 Enzyme-responsive nanoparticles

Principally, enzyme-responsive nanoparticles are sensitive to specific enzyme activity that enables high sensitivity for the specific substrates, as expected to modulate a drug release via dissolution and/or degradation of the matrix (Guo et al., 2012; Boyuklieva et al., 2025). Recently, extensive studies on enzyme-responsive systems have emerged, driven by advances in incorporating various enzyme-cleavable peptides or linkers into nanoparticles and by targeting multiple enzymes, including acetylcholinesterase, matrix metalloproteinases, and lysosomal enzymes. These strategies enable enzyme-mediated disassembly of nanoparticles and subsequent drug release at pathological lesion sites, particularly in the context of enzyme upregulation in neurodegenerative diseases (Gao et al., 2025).

Overexpression of cholinesterase is observed in AD-related pathology, resulting from a chronic cellular stress event (Toiber and Soreq, 2005). This inspires the development of a supramolecular binary vesicle, which is composed of *p*-sulfonatocalix[4]arene and enzyme-cleavable myristoylcholine that undergoes enzyme-induced disassembly triggered by the cholinesterase, thereby enabling *in vitro* controlled release of the AD drug tacrine and demonstrating potential for targeted drug delivery (Guo et al., 2012).

##### 4.2.4.5 Magnetic field-responsive nanoparticles

Magnetic field-responsive nanoparticles have drawn widespread interest in recent advanced brain stimulation approaches to the treatment of AD, as they offer non-invasive, precise, and targeted drug delivery (Giménez et al., 2024). Owing to their ability to respond to magnetic fields, the therapeutic potential of magnetic nanoparticles can be enhanced through controlled drug release, thereby supporting their promise as a novel platform for both the diagnosis and treatment of AD (Chaparro et al., 2023). Osmotin-loaded magnetic nanoparticles have demonstrated successful BBB penetration when guided by functionalized magnetic fields. This approach results in nanoparticle accumulation in the cortex and hippocampus of mice after intravenous injection without inducing neurotoxicity or compromising BBB integrity and ultimately leads to attenuated synaptic deficits and tau hyperphosphorylation (Amin et al., 2017).

To provide a concise overview of the stimuli-responsive nanoparticle platforms discussed above, Table 1 summarizes their key strengths and weaknesses with respect to AD drug delivery. This comparative assessment serves as a practical reference for selecting or designing nanoparticle systems tailored to specific therapeutic strategies and pathological targets in the treatment of AD.

**Table 1:** Comparative strengths and weaknesses of various drug delivery platforms for Alzheimer's disease drug delivery.

Drug delivery platform	Brain targeting/ BBB penetration	Drug loading capacity	Stability/ shelf-life	Clinical readiness/ scalability	Strength/ weakness
Lipid-based nanoparticles					
SLNs	++	++	+	++	High versatility, scalable fabrication, needs optimization for BBB (Satapathy et al., 2021; Bian et al., 2024).
NLCs	+++	+++	+++	++	Superior loading, reduced leakage, nose-to-brain delivery possible (Duan et al., 2020; Ali et al., 2024).
Liposomes	+++	++	+	+	Surface functionalization for brain-targeting, protective bilayer (Arora et al., 2020; Hernandez and Shukla, 2022).
Exosomes	+++	++	+	+	Excellent biocompatibility and BBB penetration, difficult purification (Pan et al., 2020; Ebadpour et al., 2025).
Polymeric-based nanoparticles					
PLA/PLGA NPs	++	+	++	++	FDA-approved polymers, biodegradable, needs engineering for BBB targeting (Kumar and Kumar, 2019; Lu et al., 2023).
Chitosan NPs	+++	+	+	+	Mucoadhesive, excellent intranasal delivery, environmentally unfriendly production (Abd Elgadir et al., 2015; Gothwal et al., 2023).
Protein NPs	+++	++	++	+	Self-assembling, versatile cargo (Yonesi et al., 2021; Bhattacharya et al., 2022; Roamcharern et al., 2025).
Dendrimers	++	++	++	+	Highly branched, precise loading, mucosal penetration, costly synthesis (Pérez-Carrión and Posadas, 2023; Suthar et al., 2025).
Other					
ADCs	+++	+	+	+	High specificity, limited half-life, costly synthesis (Feng and Wang, 2025; Kim et al., 2025).
pH-Responsive	++	++	+	+	Acid-triggered release, enhanced endosomal escape (Cai et al., 2020).
ROS-Responsive	++	++	+	+	Controlled release at oxidative stress sites, anti-Aβ aggregation (Wang et al., 2024; Chen et al., 2025).
temperature-responsive	++	++	+	+	Triggered release via photothermal effect, precise BBB targeting (Lai et al., 2020; Zhou et al., 2020).
Enzyme-responsive	++	++	+	+	Specific release at enzyme-upregulated sites, complex design (Guo et al., 2012; Gao et al., 2025).
Magnetic field-responsive	++	+	+	+	Non-invasive, guided delivery, requires external equipment (Amin et al., 2017; Chaparro et al., 2023).

Nanoparticle platforms were qualitatively compared using a three-level scoring system designed to reflect comparative trends reported in the included literature. Scoring system: + denotes limited performance or early-stage evidence, ++ denotes moderate performance, and +++ denotes strong performance or translational evidence.

### 4.3 Systemically relevant effects of nanoparticles

The application of nanoparticles for drug delivery highlights key questions regarding their *in vivo* behavior, particularly their interactions with biological fluids and interfaces. Several biologically relevant challenges have been reported; however, key issues such as the corona effect, shear stress effect, and intracellular trafficking process will be discussed in this section to further illustrate the difficulties associated with translating nanoparticles for drug delivery in the treatment of AD.

The term “protein corona” describes the layer of proteins adsorbed onto the particle surface, which is classified into the hard protein corona—tightly bound proteins with higher affinity—and the soft protein corona—loosely bound proteins with higher exchange rates (Winzen et al., 2015). To date, the term “bio-corona” has been introduced as an alternative to “protein corona” to more accurately represent the complex array of biomolecular components present in biological fluids to which nanoparticles are exposed (Fadeel, 2022). The corona effect arises when functionalized nanoparticles enter the bloodstream and interact with biological molecules that adsorb onto their surfaces. In this context, understanding how the corona influences the *in vivo* biological properties of nanoparticles remains a major challenge for the rational design of nanomaterials for clinical applications. It is important to note that, unlike healthy individuals, patients with AD exhibit a distinct protein profile among those adsorbed onto nanoparticle surfaces across various platforms (Corbo et al., 2021). While this may enhance diagnostic performance (Hadjidemetriou et al., 2021; Corbo et al., 2021), it warrants further investigation, as it may also affect the efficacy of drug delivery in therapeutic applications.

Tang et al. demonstrated that the composition of the protein corona formed on PEG-PLA nanoparticles varies depending on dosing frequency and loading conditions. Although PEGylation is generally intended to reduce protein corona formation, specific corona constituents were still observed following intravenous administration in C57BL/6 mice over 7 days. In particular, the enrichment of RAB5A and CD36 was reported to play a significant role in cellular internalization, thereby supporting nanoparticle delivery to the brain and enhancing microglial targeting efficiency. Notably, the α-mangostin-loaded formulation resulted in a greater extent of protein corona formation, likely due to changes in nanoparticle physicochemical properties, which in turn increased RAB5A and CD36 adsorption and significantly improved brain distribution and microglial uptake. Furthermore, repeated administration led to notable alterations in protein corona composition, with multiple dosing promoting increased adsorption of immunoglobulins and complement proteins (Tang et al., 2022). Modulating the protein corona provides an alternative strategy to enhance brain-targeted nanomedicine beyond conventional surface chemistry approaches; however, achieving an optimal balance between targeting efficacy and potential side effects remains a key challenge in nanoparticle design. Guerrero et al. demonstrated that tuning the protein corona to form an ApoE-enriched hard corona significantly enhances gold nanoparticle biological performance, particularly by increasing brain accumulation in Sprague-Dawley rats; however, species-specific differences may limit translation, and studies using fully humanized BBB models are needed to improve clinical relevance (Guerrero et al., 2026).

Apart from the protein corona effect, endothelial cells in the bloodstream are continuously exposed to shear stress; therefore, understanding nanoparticle behavior and nanoparticle-endothelial cell interactions under flow conditions following intravenous administration is essential (Cicha, 2016). Studies in Tg(fli1a:EGFP)y1/+ zebrafish larvae and C57BL/6 mouse models indicate that high shear stress is a critical factor in the cytotoxicity of mesoporous silica nanoparticles, particularly regarding cardiovascular toxicity in zebrafish and subacute toxicity in mice. In addition to morphological properties, spherical silica nanoparticles were shown to induce oxidative damage at accumulation sites in mice (e.g., liver, spleen, and lungs), causing more severe effects than rod-shaped silica nanoparticles. One possible explanation is that higher shear stress from faster blood flow increases interactions with vascular endothelial cells, leading to cytotoxicity and cardiovascular toxicity *in vivo* (Cheng et al., 2023). Meanwhile, low shear stress, as studied in zebrafish and C57BL/6 mouse models, promotes oxidative stress-mediated uptake of red blood cell-derived extracellular vesicles (RBCEVs) by brain endothelial cells (Qin et al., 2022). These highlight the shear stress- and shape-dependent mechanisms of nanoparticle uptake and toxicity.

The endo-lysosomal pathway is a major bottleneck limiting the clinical efficacy of therapeutic agents, as nanoparticles are often sequestered and degraded following endocytosis, reducing bioavailability (Ahmad et al., 2019). A study on ROS-responsive biomimetic exosome-liposome hybrid nanovesicles (TSEL) demonstrated promising efficacy in overcoming the endosomal/lysosomal barrier *in vitro*, as evidenced by the dispersion of gene cargo around the nucleus and a transient peak in the colocalization coefficient followed by a decrease, indicating successful endosomal escape. Furthermore, TSEL nanovesicles co-delivering BACE1 siRNA and a TREM2 plasmid effectively penetrated the BBB and accumulated at AD lesions *in vivo* in APP/PS1 mice (Jiang et al., 2024). Liu et al. demonstrated the critical role of endosomal escape in achieving therapeutic efficacy, showing that effective dissociation of DNA and peptide cargo is necessary for their respective actions in the cytoplasm and nucleus. This process is facilitated by the protonation of amine groups grafted onto the polymer surface within endosomes (pH 5–6) and lysosomes (pH 4–5), which possess strong proton-buffering capacity and induce rapid osmotic swelling, thereby promoting cargo release. *In vivo*, endosomal escape enabled the released therapeutic gene (pBACE1-AS) to downregulate BACE1 expression, resulting in reduced extracellular Aβ plaque formation, while d-peptides concurrently inhibited tau fibrillation, decreasing intracellular tau aggregates (Liu et al., 2016). Taken together, these studies underscore the significant challenges associated with translating nanoparticles into clinical applications, highlighting the need for these limitations to be more rigorously addressed in the design and development of drug delivery strategies.

### 4.4 Emerging advanced strategies for Alzheimer's treatment

#### 4.4.1 Receptor-mediated transcytosis (RMT)

Receptor-mediated transcytosis (RMT) is a highly selective cellular transport mechanism that enables macromolecules to traverse the BBB via vesicular trafficking. Unlike passive diffusion, which is largely restricted to small lipophilic compounds, RMT exploits endogenous receptors expressed on brain endothelial cells, allowing the transport of biologics and engineered nanoparticles that would otherwise be excluded from the CNS (Fullstone et al., 2016).

**Figure 4:**
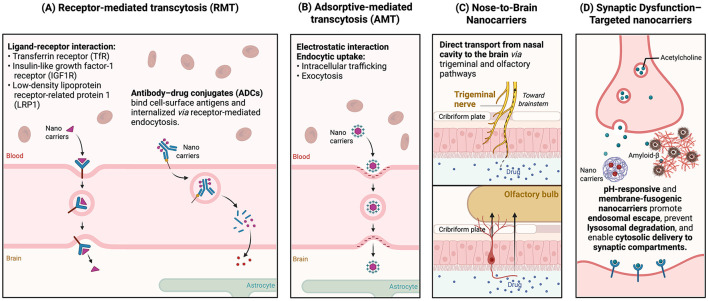
Advanced nanoparticle-mediated transport strategies for targeted drug delivery in Alzheimer's disease. Schematic illustration of key nanoparticle-based mechanisms for enhancing therapeutic transport to the brain. **(A)** Receptor-mediated transcytosis (RMT) utilizes ligand-receptor interactions–such as transferrin, insulin-like growth factor-1, and low-density lipoprotein receptor-related protein 1–to facilitate vesicular transport across the blood–brain barrier (BBB). Antibody-drug conjugates (ADCs) bind cell-surface antigens, enabling a receptor-mediated internalization and targeted intracellular drug release. **(B)** Adsorptive-mediated transcytosis (AMT) relies on electrostatic interactions between charged nanoparticles and endothelial membranes to promote endocytic uptake and transcellular trafficking. **(C)** Nose-to-brain nanoparticles may help overcome some BBB-related delivery barriers through olfactory and trigeminal pathways, depending on the properties of the cargo and formulation. **(D)** Synaptic dysfunction-targeted nanoparticles employ stimuli-responsive and membrane-fusogenic properties to enhance endosomal escape, prevent lysosomal degradation, and enable cytosolic delivery to synaptic compartments. Figure created with BioRender.com and adapted from the “Direct Pathways of Nose-to-Brain Biomolecule Delivery” template by Mina Nashed (BioRender). Licensed under CC BY 4.0 (https://creativecommons.org/licenses/by/4.0/).

Principally, RMT involves ligand binding to receptors on the luminal endothelial surface, followed by receptor-mediated endocytosis, intracellular trafficking, and exocytosis into the brain parenchyma. Internalization typically occurs through clathrin- or caveolin-dependent pathways, with actin-mediated processes supporting vesicular transport (Baghirov, 2025) (Figure 4A). A defining feature of RMT is its high specificity driven by ligand-receptor interactions, enabling the uptake of large macromolecules, nanoparticles, and biologics (Choi and Shusta, 2023). The process is energy-dependent and may support bidirectional transport, which is particularly relevant in AD, where RMT pathways contribute not only to CNS drug delivery but also to the clearance of pathological proteins such as Aβ (Pflanzner et al., 2010). These properties position RMT as a suitable approach for therapeutics requiring precise targeting and sustained brain exposure.

The selection of appropriate receptors is a critical determinant of delivery efficiency (Zhang et al., 2020; Haqqani et al., 2024). The TfR remains the most extensively studied target due to its abundant expression at the BBB and its well-characterized transcytosis pathway, and it has been widely leveraged to enhance CNS delivery of antibodies and enzyme inhibitors relevant to AD (Shen et al., 2025). However, the performance of TfR-targeting strategies may vary depending on aging, disease stage, and neurovascular alterations, which may influence BBB biology and alter receptor-mediated transport (Petralla et al., 2024b; Torres et al., 2026). Accordingly, the efficacy of TfR-based delivery systems should be interpreted in a context-dependent manner rather than assumed to be consistent across all patients with AD. The insulin receptor and insulin-like growth factor-1 receptor (IGF1R) have also attracted interest owing to their rapid transport kinetics and roles in neuronal metabolism (Haqqani et al., 2024). Meanwhile, low-density lipoprotein receptor-related protein 1 (LRP1) is particularly relevant to AD pathophysiology because of its involvement in lipid trafficking and amyloid-β clearance, offering the dual advantage of facilitating drug transport while potentially influencing disease mechanisms (Petralla et al., 2024a).

Ligand density and binding affinity further govern transcytosis efficiency. Moderate-affinity interactions generally promote productive transport, whereas excessively strong binding may result in receptor retention and lysosomal degradation rather than BBB passage (Kochman et al., 2026). In particular, in TfR-targeted systems, excessively high-affinity binding may create a binding-site barrier, leading to preferential endothelial retention and intracellular sequestration rather than productive transcytosis across the BBB (Bien-Ly et al., 2014). Likewise, although surface functionalization can enhance receptor targeting, excessive ligand density does not necessarily improve transport efficiency and may instead cause steric modification, alter receptor engagement, or accelerate systemic clearance. Receptor saturation at high systemic doses can further reduce transport efficiency and increase peripheral sequestration (Pinkiewicz et al., 2025), while lysosomal trapping may degrade internalized cargo before abluminal release, while lysosomal trapping may degrade internalized cargo before abluminal release (Salimi et al., 2023). Additionally, receptor expression and transcytosis capacity may be altered by aging and neurodegeneration, contributing to interpatient variability. Species-specific differences in receptor distribution further complicate extrapolation from preclinical models to humans, and the presence of these receptors in peripheral tissues raises concerns regarding off-target uptake (Zhang et al., 2020). Taken together, a careful optimization of ligand chemistry, density, and surface presentation is essential for maximizing CNS delivery.

In antibody-based CNS delivery, conventional antibodies have limited intrinsic BBB penetration. To overcome this constraint, CNS-directed strategies often employ receptor-mediated transcytosis through engineered formats such as bispecific antibodies or brain-shuttle constructs targeting BBB receptors (e.g., TfR or insulin receptor) together with pathological targets such as Aβ or tau, thereby improving brain delivery compared with conventional antibodies (Dixit et al., 2025). In this context, ADCs may further extend this approach by linking therapeutic payloads to BBB-targeting antibody scaffolds for more selective CNS delivery. Nanoparticle-based systems likewise provide a versatile RMT platform, as polymeric, lipid-based, and hybrid nanoparticles can be functionalized with receptor-targeting ligands while encapsulating diverse therapeutics (Terstappen et al., 2021). In AD models, these platforms have been explored for the delivery of anti-amyloid agents, tau-directed therapeutics, anti-inflammatory drugs, and gene-based therapies (Stanimirovic et al., 2018; Chen et al., 2024a; Pan et al., 2025; Sehlin et al., 2025).

#### 4.4.2 Adsorptive-mediated transcytosis (AMT)

Unlike RMT, Adsorptive-mediated transcytosis (AMT) does not rely on specific ligand-receptor interactions but instead exploits the net surface charge of therapeutic agents or delivery systems to promote nonspecific adsorption, endocytosis, and subsequent vesicular transport across the BBB (Herve et al., 2008; Puri et al., 2024). At the molecular level, AMT is initiated by the electrostatic attraction between cationic molecules and negatively charged components of the endothelial glycocalyx and plasma membrane. This interaction triggers endocytic uptake, followed by intracellular trafficking and exocytosis at the abluminal membrane into the brain parenchyma. A wide range of cationic delivery systems, including cationized proteins, cell-penetrating peptides (CPPs), liposomes, polymeric nanoparticles, dendrimers, and solid lipid nanoparticles, have been engineered to exploit this pathway (Alo and Lamers, 2025). Compared with RMT, AMT offers a relatively high transport capacity and is less constrained by receptor availability, making it attractive for delivering large or structurally diverse therapeutic cargos (Figure 4B).

In the context of AD, AMT has been explored as a strategy to enhance the delivery of therapeutic agents with inherently poor BBB permeability. For example, nonsteroidal anti-inflammatory drugs such as flurbiprofen, which exhibit limited CNS penetration in their free form, have shown improved brain uptake when coupled with cationic carriers that utilize AMT (Al-Azzawi et al., 2020). Similarly, various nanoparticle systems—including polymeric nanoparticles, lipid nanoparticles, and dendrimers—have been developed to deliver anti-amyloid, anti-inflammatory, and neuroprotective agents across the BBB via AMT, thereby improving their therapeutic potential in AD models (Bhattacharya, 2023; Mazahir and Yadav, 2023).

Despite its potential, AMT-based drug delivery faces several challenges. The nonspecific nature of electrostatic interactions raises concerns regarding off-target uptake in peripheral tissues, reduced brain selectivity, and systemic exposure. Moreover, excessive cationic surface charge can induce cytotoxicity, immunogenicity, and membrane disruption, compromising BBB integrity and neuronal safety (Wang et al., 2023a). The instability of cationic proteins and CPP-based systems in biological fluids further complicates their clinical translation. In addition, BBB impairment in AD may unpredictably influence AMT efficiency, leading to variability in therapeutic outcomes (Herve et al., 2008).

To this end, contemporary DDS increasingly incorporates surface charge engineering strategies aimed at balancing transport efficiency and safety. These approaches include partial charge shielding, stimuli-responsive surface activation, and the use of biodegradable or zwitterionic materials to reduce nonspecific interactions. Hybrid delivery platforms that combine AMT with receptor-mediated transcytosis have also been developed to improve selectivity and control while retaining the high transport capacity of electrostatic interactions (Tashima, 2022; Pan et al., 2025; Sehlin et al., 2025). Such combinatorial strategies may offer a more refined approach to BBB penetration in AD.

#### 4.4.3 Nose-to-brain delivery

Intranasal drug delivery has been recognized as a promising non-invasive strategy for CNS targeting in AD because it may enable nose-to-brain transport through olfactory and trigeminal pathways. This route can promote rapid brain exposure while potentially reducing systemic distribution and first-pass metabolism, thereby addressing an important limitation of conventional systemic therapies (Fonseca et al., 2021). Mechanistically, intranasally administered agents may be absorbed through the olfactory epithelium or trigeminal nerve endings and transported to the brain via extracellular diffusion and intracellular axonal pathways, which may partially bypass BBB-related constraints (Raghav et al., 2023; Li et al., 2025; Wu et al., 2025). Detectable CNS concentrations have been observed within minutes, suggesting a potentially faster onset than peripheral administration under specific experimental conditions (Hanson and Frey, 2008) (Figure 4C).

Key advantages of intranasal delivery include (i) enhanced brain bioavailability, (ii) reduced systemic toxicity, and (iii) improved patient compliance, particularly for chronic neurodegenerative disorders requiring long-term therapy (Hanson and Frey, 2008; Formica et al., 2022). This approach is especially suitable for cargos with poor BBB permeability, such as peptides, proteins, nanoparticles, and EVs. For instance, intranasal insulin has demonstrated neuroprotective effects and improved cognitive outcomes in patients with mild cognitive impairment and AD, highlighting the translational potential of this strategy (Hanson and Frey, 2008). Similarly, intranasal antibodies targeting Aβ have reduced cerebral amyloid burden and improved cognition in preclinical models (Xiao et al., 2013). However, within a nasal cavity, a rapid mucociliary clearance and enzymatic degradation play an important role in reducing drug stability and brain uptake, while interindividual variability in nasal anatomy and epithelial permeability may contribute to inconsistent therapeutic responses (Fan et al., 2018; Wong et al., 2024). In addition, restricted dosing volumes and the need for repeated administration may affect long-term treatment adherence (Fonseca et al., 2021). For chronic conditions such as AD, repeated intranasal dosing may also raise concerns regarding local mucosal irritation, inflammatory responses, epithelial injury, or impaired mucociliary function (Keller et al., 2022; Chen et al., 2024b). Accordingly, long-term nasal safety and tolerability should be carefully considered in the translational evaluation of nose-to-brain delivery systems.

To address these challenges, advanced formulations have been developed to enhance nasal residence time and transport efficiency, such as nanoparticles, liposomes, and EVs, which can protect therapeutic agents from degradation and facilitate neuronal delivery (Fan et al., 2018; Jiang et al., 2023; Li et al., 2025). Additionally, incorporation with mucoadhesive polymers or targeting ligands further improves retention and brain targeting, while permeation enhancers may increase epithelial absorption, although safety considerations remain critical (Fonseca et al., 2021).

#### 4.4.4 Targeting synaptic dysfunction

Synaptic dysfunction is widely recognized as one of the earliest and strongest correlates of cognitive decline in AD. Soluble Aβ oligomers, pathological tau, and neuroinflammatory mediators disrupt synaptic plasticity, impair neurotransmission, and alter dendritic spine architecture long before extensive neuronal loss occurs. Notably, synapse loss correlates more closely with cognitive impairment than Aβ plaque burden, supporting the concept that AD is fundamentally a disorder of synaptic failure (Selkoe, 2002). Experimental studies further demonstrate that Aβ oligomers directly inhibit long-term potentiation and memory formation, highlighting synapses as critical early therapeutic targets (De Strooper and Karran, 2016). Consequently, delivery strategies capable of directly modulating synaptic compartments may offer opportunities for early disease intervention.

Targeting synapses remains challenging due to their highly compartmentalized structure and dynamic regulation across complex neuronal networks. Advanced DDS are therefore being engineered to achieve a precise cellular and subcellular targeting. Nanoparticles functionalized with neuron-specific ligands can promote preferential neuronal uptake and increase drug accumulation at synaptic sites while minimizing systemic exposure. Peptides derived from rabies virus glycoprotein and tetanus toxin fragment C have been extensively utilized to enhance neuron-selective internalization of nanoparticles and biologics across the BBB (Townsend et al., 2007; Liu et al., 2009) (Figure 4D).

Following cellular uptake, efficient intracellular trafficking is essential for therapeutic efficacy. pH-responsive polymeric nanoparticles, membrane-fusogenic lipid carriers, and other endosomal escape strategies have been developed to prevent lysosomal degradation and enable cytosolic delivery of therapeutic cargos (Karlsson et al., 2019). Delivery of exosomes loaded with siRNA, neurotrophic factors, or anti-inflammatory agents has resulted in efficient neuronal uptake, enhanced synaptic plasticity, and improved memory in AD models. Their low immunogenicity and inherent BBB permeability position them as promising candidates for synapse-directed therapy (Alvarez-Erviti et al., 2011; Xin et al., 2013; Reza-Zaldivar et al., 2019). Clinical studies of intranasal insulin have shown cognitive improvement and enhanced functional connectivity, indicating beneficial effects on synaptic activity (Craft et al., 2020). Interestingly, intranasal nanoparticle systems have also demonstrated increased hippocampal drug accumulation and restoration of synaptic plasticity in neurodegenerative models (Ong et al., 2014).

Nevertheless, significant translational challenges remain. Targeting ligands must achieve high specificity without interfering with physiological synaptic signaling, while intracellular delivery systems must balance endosomal escape with minimal cytotoxicity. Moreover, prolonged modulation of synaptic pathways carries potential risks of excitotoxicity and maladaptive plasticity, emphasizing the need for tightly controlled release kinetics. The development of biomarkers and imaging tools capable of confirming synaptic target engagement *in vivo* will be critical for successful clinical translation (Chen et al., 2019; Jackson et al., 2019).

## 5 Clinical trials and translational progress

Recent clinical advances in AD therapeutics highlight both the progress achieved and the persistent challenges associated with effective drug delivery to the central nervous system. One of the most extensively investigated strategies that directly addresses BBB constraints is intranasal insulin delivery. Several clinical studies have evaluated intranasal insulin in individuals with cognitive impairment or early AD, with some reporting improvements in memory performance and metabolic brain integrity (Hallschmid, 2021; Silva et al., 2025). For example, intranasal insulin has been shown to enhance memory and reduce progression of white matter hyperintensity volume over 12 months, supporting its potential as a BBB-bypass therapeutic via olfactory and trigeminal pathways (Kellar et al., 2021). However, larger randomized controlled trials have produced mixed findings, with some reporting no significant cognitive or functional benefit compared with placebo, emphasizing the need for optimized dosing strategies, longer follow-up, and biomarker-guided study designs (Craft et al., 2020).

Beyond BBB-bypass strategies such as intranasal delivery, major regulatory milestones in AD have been achieved with anti-amyloid monoclonal antibodies. Aducanumab received accelerated U.S. FDA approval in 2021 based on its ability to reduce cerebral amyloid plaques, although its clinical benefit remains controversial due to inconsistent Phase III outcomes (Sevigny et al., 2016; Dhruva et al., 2023; Ashique et al., 2024). More recently, lecanemab and donanemab have demonstrated clearer evidence of amyloid reduction with modest slowing of cognitive decline, leading to regulatory approvals in several regions (Mintun et al., 2021; Van Dyck et al., 2023). However, their clinical utility is constrained by inefficient BBB penetration, necessitating repeated high-dose intravenous administration. Safety concerns, particularly amyloid-related imaging abnormalities (ARIA), remain important considerations despite generally manageable risk profiles (Withington and Turner, 2022; Elamin et al., 2025).

Collectively, these clinical experiences highlight a key translational challenge: even when therapeutic targets are biologically validated, inefficient CNS delivery limits treatment efficacy while increasing systemic burden. This has accelerated efforts to develop next-generation DDS capable of enhancing BBB penetration, improving brain-specific targeting, and reducing peripheral exposure.

Nanotechnology-based carriers, receptor-targeted delivery platforms, intranasal nanoparticle systems, and extracellular vesicle-based formulations have demonstrated improved pharmacokinetics, controlled release, and enhanced CNS accumulation in preclinical studies, with several advanced platforms now progressing into early-phase clinical evaluation for AD and related neurodegenerative disorders. Among the most clinically advanced biomimetic delivery systems, allogeneic human adipose mesenchymal stromal cell-derived exosomes (MSC-EVs) have entered Phase I/II clinical trials in patients with mild to moderate AD, where repeated intranasal administration was shown to be safe and well tolerated, with preliminary evidence of cognitive stabilization and reduced hippocampal atrophy. These findings represent one of the first clinical demonstrations of extracellular vesicles functioning as naturally derived nanoparticles for CNS drug delivery (Xie et al., 2023).

Another emerging strategy involves receptor-targeted BBB-shuttle biologics. Trontinemab, an anti-amyloid monoclonal antibody fused to a transferrin receptor-binding shuttle module designed to enhance receptor-mediated transcytosis, is currently being evaluated in Phase I/II clinical studies, with early data indicating substantially increased brain exposure compared with conventional antibodies (Grimm et al., 2023). Although biologic in structure, such constructs embody advanced delivery principles by integrating receptor-targeted transport mechanisms to overcome BBB limitations. Together, these early clinical advances signal a transition from theoretical delivery strategies toward translationally viable DDS for AD.

It is worth noting that an important limitation in the translation of preclinical findings to humans is the heavy reliance on transgenic mouse models, particularly APP/PS1 and 5xFAD. Although these models are valuable for studying amyloid and tau pathology, they do not fully recapitulate the complexity of human AD or the pharmacokinetic behavior of the human BBB (Onos et al., 2016). More broadly, genetic mouse models of AD have shown only limited success in predicting clinical outcomes, underscoring the need for caution when extrapolating preclinical efficacy to human disease (Alexandra Lopes and Guil-Guerrero, 2025). In addition, species differences in BBB transport systems, including efflux transporters such as P-gp and targeting receptors such as the transferrin receptor, represent an important source of translational uncertainty, as their abundance and activity may differ between rodents and humans (Syvanen et al., 2009; Verscheijden et al., 2021; Yesiltepe et al., 2026). These differences are particularly relevant for nanoparticle-associated cargos and receptor-targeted delivery systems, as transporter kinetics and receptor availability observed in mouse models may not accurately predict human brain penetration, retention, or therapeutic response. Dose translation remains a major challenge, because extrapolating nanomaterial pharmacokinetics from preclinical animals to humans can be confounded not only by body-size relationships, but also by interspecies differences in anatomy, physiology, immunology, and mononuclear phagocyte system function. This challenge is particularly relevant for nanoparticle-based delivery systems, whose disposition is often formulation-dependent and may involve prolonged circulation, nonlinear pharmacokinetics, and extensive tissue distribution that are not adequately captured by conventional scaling assumptions (Valic and Zheng, 2019). Therefore, findings derived from AD transgenic mouse models should be interpreted with caution and, where possible, complemented by human-relevant BBB models and translational pharmacokinetic evaluation.

An additional clinical concern is whether heterogeneous BBB leakage in AD could lead to disproportionate nanoparticle accumulation in regions of increased permeability, thereby increasing the risk of local neurovascular toxicity (Alkhalifa et al., 2023). Although no validated dosing algorithm currently exists for this scenario, the issue highlights the need for cautious translational development. Because BBB dysfunction in AD varies across patients, disease stages, and vascular comorbidities, and genetic factors, fixed dosing strategies may not produce uniform brain exposure. In particular, APOE ε4 has been linked to greater BBB dysfunction in AD and may further contribute to interpatient variability in brain exposure and treatment safety (Montagne et al., 2020). Future nanoparticle studies may therefore need to consider risk-adapted approaches, including lower starting doses, gradual dose escalation, patient stratification based on genetic, vascular and imaging biomarkers, and serial neuroimaging surveillance (Lee et al., 2026). Another important translational concern is nanoparticle immunogenicity, particularly with repeated dosing in chronic diseases such as AD. Liposomal and PEGylated nanoparticles may trigger complement activation-related pseudoallergy (CARPA), hypersensitivity reactions, or accelerated blood clearance, which can affect both safety and treatment consistency. In addition, impaired glymphatic or paravascular clearance in AD may alter the elimination of brain-delivered nanoparticles, potentially affecting retention, regional distribution, and long-term safety during repeated dosing (Gu et al., 2020; Dobrovolskaia, 2022). These issues should also be considered during nanoparticle design and preclinical evaluation. More broadly, the need for careful safety monitoring is also underscored by experience with anti-amyloid therapies, in which ARIA the risk of amyloid-related imaging abnormalities (ARIA) has been associated with drug dose, APOE ε4 status, baseline microhemorrhages, and other vascular risk factors (Barakos et al., 2022; Doran and Sawyer, 2024).

## 6 Opportunities, challenges, and future directions

In recent years, nanoparticles have emerged as a promising strategy in AD therapy, offering considerable potential for precise drug delivery while presenting challenges that must be carefully addressed by researchers. To the best of our knowledge, nanoparticles offer significant opportunities for clinical translation in AD therapy. Their advanced nanoparticles approaches are particularly promising, including: (i) enhanced BBB penetration—engineered to overcome the BBB via various mechanisms, paving the way for effective CNS delivery (Bian et al., 2024; Du et al., 2024); (ii) precise and targeted drug delivery—enabling drugs to reach pathological sites specifically while minimizing systemic exposure and toxicity (Wang et al., 2021; Cai et al., 2023; Sairam et al., 2023); (iii) controlled drug release—allowing sustained and stimuli-responsive release, ultimately improving therapeutic efficacy (Natarajan et al., 2014; Adepu and Ramakrishna, 2021; Askarizadeh et al., 2023); and (iv) personalized medicine potential—supporting patient-specific targeting or combination therapies (Dey et al., 2024).

Nevertheless, several important challenges continue to limit the clinical translation of nanoparticles in AD therapy. Safety remains a major concern, particularly with respect to long-term exposure, immunogenicity, and the accumulation of nanomaterials in the brain or peripheral organs (Gao et al., 2026). Although the use of biodegradable materials may help reduce some of these risks, safety profiles must be evaluated carefully for each platform. In addition, BBB transport remains variable and often inefficient because of the structural and functional complexity of the barrier, as well as the heterogeneous nature of BBB alterations across patients and disease stages in AD (Gireud-Goss et al., 2021; Wu et al., 2023). This challenge is further compounded by disease-associated BBB dysfunction that does not occur uniformly throughout disease progression. As highlighted by Alkhalifa et al. (2023), BBB breakdown in AD may arise early and is influenced by multiple pathological processes, including vascular dysfunction, neuroinflammation, and cerebral amyloid angiopathy, resulting in regional and inter-individual variability in barrier permeability (Alkhalifa et al., 2023). Such heterogeneity has important implications for nanoparticle-based drug delivery, as differences in BBB integrity may affect brain exposure, therapeutic response, and the risk of off-target or neurovascular toxicity. Therefore, BBB-targeted delivery strategies in AD should be evaluated not only for their capacity to enhance brain transport, but also for their performance across heterogeneous disease states that may influence dosing consistency, safety, and translational predictability. As a result, rational design strategies and disease-relevant evaluation models remain necessary to improve delivery consistency and predict therapeutic outcomes. Manufacturing challenges further complicate translation, including issues related to reproducibility, quality control, scale-up, and regulatory assessment (Lu et al., 2023; Na et al., 2023). The temporal progression of AD should also be considered in BBB-targeted drug delivery. Early and late stages differ in BBB integrity, neurovascular dysfunction, neuroinflammatory status, and the expression or function of transport-related molecules such as LRP1 and RAGE, all of which may influence nanoparticle performance and delivery consistency (Deane, 2012; Chen Y. et al., 2023; Petralla et al., 2024a). Therefore, nanoparticle-based strategies should be evaluated in a stage-aware manner to improve translational relevance and therapeutic predictability. A further point to consider is nanoparticle degradation during storage and after administration. Lipid-based systems may undergo oxidation or hydrolysis, whereas polymeric carriers may undergo hydrolytic or enzymatic degradation. These processes can affect formulation stability, drug release, and safety, while the resulting degradation products may also contribute to toxicity during chronic administration (Perrigue et al., 2021).

Importantly, commonly used *in vitro* BBB models, such as transwell systems and endothelial cell lines including RBE4, bEnd.3, and hCMEC/D3 monolayers, have important limitations, as they may overpredict permeability and do not fully recapitulate key *in vivo* BBB features, including physiological shear stress and multicellular neurovascular architecture (Chaulagain et al., 2023). Because shear stress can influence endothelial phenotype as well as nanoparticle adhesion and uptake at the BBB, static systems may have limited translational relevance when evaluating targeting efficiency (Katt and Shusta, 2020). Moreover, increased brain uptake *in vivo* does not necessarily indicate true parenchymal penetration, as part of the detected signal may reflect vascular-associated accumulation. Therefore, complementary validation approaches, including capillary depletion analysis and confocal imaging with endothelial markers, are important for improving the rigor and translational interpretation of preclinical BBB delivery studies (Van Rooy et al., 2011). In addition, magnetic resonance-guided focused ultrasound (MRgFUS) has emerged as a promising non-invasive strategy for transient BBB opening. In combination with systemically administered microbubbles, it enables localized and reversible BBB disruption, thereby enhancing drug delivery to targeted brain regions. In AD, early clinical studies support the feasibility of this approach and suggest a favorable short-term safety profile. MRgFUS therefore represents a potentially useful adjunct platform for improving the brain delivery of biologics and other disease-modifying therapies, although further studies are needed to establish its long-term safety and clinical efficacy (Rezai et al., 2022). Another important consideration is the quantitative interpretation of nanoparticle performance. Brain uptake alone may not adequately reflect delivery efficiency without pharmacokinetic context. Brain-to-plasma exposure metrics, such as *K*_p, brain_, may provide a useful measure of total brain exposure relative to systemic exposure, while *K*_p, uu, brain_, where feasible, may offer a more informative assessment of unbound brain exposure at the BBB. In addition, compartmental or PBPK-based modeling may improve the interpretation of nanocarrier distribution, particularly for systems with formulation-dependent or nonlinear pharmacokinetics (Yuan et al., 2019; Ma et al., 2024). In addition, the lack of a well-defined regulatory framework remains a major challenge for nanoparticle-based AD therapies, limiting their clinical translation; therefore, the development and implementation of clear and effective regulatory guidelines are essential. The FDA identified nanotechnology as a key component of its Critical Path Initiative, which aims to modernize the medical product development process (Sadrieh and Espandiari, 2006). Beyond the FDA's regulatory pathway, other standards-setting organizations, such as the European Medicines Agency (EMA), ASTM International, and the International Organization for Standardization (ISO), have also published various guidelines and standards for nanotechnology, highlighting their active involvement in advancing and harmonizing regulatory frameworks in this field (Khopade and Shah, 2025). Accordingly, regulatory pathways guided by recommendations from the FDA and other agencies should be carefully followed. These include the identification of critical quality attributes to ensure the consistent quality of nanotechnology-based medicinal products, thereby addressing batch-to-batch reproducibility. In addition, the application of risk assessment strategies, the use of appropriate and standardized characterization methods, and the evaluation of drug release profiles, particularly for complex systems such as liposomes, are essential to support the safe development, regulatory approval, and post-market monitoring of these products (Sadrieh and Espandiari, 2006; Çetintaş et al., 2021; Maheshwari et al., 2025).

Future progress in AD nanoparticles will likely depend on the development of next-generation platforms with improved translational feasibility. These may include multifunctional or stimuli-responsive systems capable of co-delivering agents targeting amyloid pathology, tau pathology, and neuroinflammation, as well as formulations optimized for less invasive administration routes such as intranasal delivery. The challenge of co-encapsulating hydrophobic and hydrophilic drugs in a single nanoparticle with controlled release should be investigated, as this strategy addresses the difficulties associated with delivering structurally dissimilar drugs. For example, composite carriers that combine micelles and liposomes offer a promising solution, allowing one drug to be loaded in the micelle core while the other is incorporated into the lipid membrane or the aqueous core, thereby enabling precise modulation of drug release kinetics (Qian et al., 2023). In addition, the integration of nanoparticles with diagnostic or monitoring tools may support more precise therapeutic stratification and facilitate theranostic applications. However, the successful translation of these approaches will require not only technological innovation, but also rigorous attention to safety, manufacturability, regulatory readiness, and clinical relevance.

In summary, nanoparticle-based systems offer considerable promise for AD therapy, particularly as platforms for improving CNS drug delivery and enabling more targeted therapeutic strategies. However, their clinical value will depend on how effectively current limitations in safety, BBB transport variability, large-scale production, and translational predictability can be addressed. To improve clarity and facilitate comparison across conceptually dense translational strategies, a comparative summary of the major therapeutic and delivery platforms discussed in Sections 5, 6 is provided in Table 2.

**Table 2:** Comparative summary of clinical progress, translational opportunities, key challenges, and future directions of BBB-oriented therapeutic strategies for Alzheimer's disease.

Strategy/theme	Principle/ translational role	Current progress/ opportunity	Key challenges/ limitations	Future direction/ implication	References
Nanotechnology-based carriers	Enhance BBB penetration, controlled release, and brain-specific delivery	Strong preclinical promise; several advanced systems are moving toward early translational evaluation	Formulation-dependent pharmacokinetics, safety concerns, immunogenicity, and variable brain exposure	Rational design matched to disease biology and therapeutic objective	Bhattacharya, 2023; Mazahir and Yadav, 2023
Intranasal nanoparticle systems (e.g., insulin)	Combine nose-to-brain delivery with nanocarrier-mediated drug protection and targeting	Attractive non-invasive strategy with translational potential	Limited clinical validation and possible variability in delivery efficiency	Optimization of formulation design and clinical evaluation for less invasive AD therapy	Hanson and Frey, 2008; Kellar et al., 2021
Extracellular vesicle-based systems (e.g., MSC-EVs)	Biomimetic, naturally derived nanocarriers for CNS delivery	Among the most advanced biomimetic platforms; early Phase I/II data suggest safety and preliminary clinical promise	Standardization, manufacturing consistency, mechanism clarification, and long-term efficacy remain unresolved	Further clinical development of EV-based delivery as a translational DDS platform	Xie et al., 2023
Receptor-targeted BBB-shuttle biologics (e.g., trontinemab)	Enhance receptor-mediated transcytosis across the BBB	Early clinical studies indicate improved brain exposure compared with conventional antibodies	Species differences in receptor biology and uncertainty in human translational predictability	Refinement of receptor-targeting strategies and clinical validation of shuttle-based biologics	Grimm et al., 2023
Clinical translation from animal models	Preclinical efficacy and delivery performance are often assessed in transgenic AD mouse models	Useful for mechanistic studies of amyloid and tau pathology	Mouse models incompletely recapitulate human AD and human BBB pharmacokinetics; species differences in transporters and receptors reduce predictability	Greater use of human-relevant BBB models and translational pharmacokinetic evaluation	Stanimirovic et al., 2018; Chen et al., 2024a; Pan et al., 2025; Sehlin et al., 2025
BBB heterogeneity in AD	BBB dysfunction varies across patients, regions, and disease stages	Recognized as a major determinant of therapeutic exposure and response	May cause inconsistent brain exposure, off-target accumulation, and neurovascular toxicity; APOE ε4 may further increase variability	Risk-adapted dosing, patient stratification, imaging biomarkers, and serial neuroimaging surveillance	Montagne et al., 2020; Barakos et al., 2022; Doran and Sawyer, 2024
Safety and immunogenicity of nanoparticles	Critical determinant of chronic-use feasibility in AD	Biodegradable systems may reduce some risks	Long-term exposure, accumulation in brain/peripheral organs, CARPA, hypersensitivity, accelerated blood clearance, and altered glymphatic clearance	Platform-specific safety profiling and long-term evaluation during repeated dosing	Gu et al., 2020; Dobrovolskaia, 2022
Stage-aware BBB-targeted delivery	Delivery performance may differ between early and late AD due to changes in BBB integrity, neuroinflammation, and transport-related molecules	Important conceptual advance for improving translational relevance	Delivery consistency may vary across disease stage and pathological context	Evaluate nanomedicines in a stage-aware manner and align design with disease progression	Deane, 2012; Chen et al., 2023; Petralla et al., 2024a; Perrigue et al., 2021
Limitations of current BBB models	*In vitro* BBB models are widely used to screen penetration and targeting	Useful for early testing	Static transwell and endothelial monolayer models may overpredict permeability and fail to reproduce shear stress and multicellular neurovascular architecture	Use more disease-relevant and physiologically representative BBB models	Katt and Shusta, 2020; Chaulagain et al., 2023
Validation of true brain penetration	Brain uptake does not always equal true parenchymal delivery	Highlights need for more rigorous interpretation of delivery studies	Vascular-associated signal may be misinterpreted as brain penetration	Combine uptake data with capillary depletion and confocal imaging using endothelial markers	Van Rooy et al., 2011
MRgFUS-assisted BBB opening	Non-invasive, localized, reversible BBB opening using focused ultrasound plus microbubbles	Emerging adjunct strategy with encouraging early feasibility and short-term safety in AD	Long-term safety and clinical efficacy remain to be established	Potential adjunct platform to enhance delivery of biologics and disease-modifying therapies	Rezai et al., 2022
Quantitative PK interpretation	Brain uptake should be interpreted in relation to systemic exposure	Metrics such as *K*_p, brain_ along with compartmental/PBPK modeling, can improve translational interpretation	Brain uptake alone may overestimate delivery efficiency; nanoparticle PK may be nonlinear and formulation-dependent	Stronger integration of PK-based metrics into nanomedicine evaluation	Yuan et al., 2019; Ma et al., 2024
Manufacturing and regulatory translation	Clinical translation depends on reproducibility, scale-up, quality control, and regulatory readiness	Regulatory agencies and standards bodies already provide relevant frameworks and standards	Lack of a fully defined regulatory pathway for complex nanotherapies	Standardized characterization, critical quality attributes, risk assessment, and batch-to-batch control	Sadrieh and Espandiari, 2006; Khopade and Shah, 2025
Next-generation nanoparticle design	Multifunctional, stimuli-responsive, or co-delivery systems may better address multifactorial AD pathology	Promising direction for targeting amyloid, tau, and neuroinflammation simultaneously	Co-encapsulation of structurally dissimilar drugs and controlled release remain technically challenging	Development of composite carriers, theranostic platforms, and clinically feasible multifunctional systems	Qian et al., 2023

## 7 Conclusions

The AD continues to pose a major global health challenge, with current treatments offering limited benefits and failing to stop disease progression. The BBB restricts effective drug delivery, but nanoparticle-based systems provide a promising solution by improving stability, targeting, and brain accumulation while reducing toxicity. Despite challenges in achieving consistent BBB penetration, several nanoparticle types, such as lipid- and polymer-based nanoparticles, show strong translational potential. Overall, nanoparticle technologies offer a hopeful path toward safer, more precise, and clinically viable therapies for AD.
